# Birth Cohort, Age, and Sex Strongly Modulate Effects of Lipid Risk Alleles Identified in Genome-Wide Association Studies

**DOI:** 10.1371/journal.pone.0136319

**Published:** 2015-08-21

**Authors:** Alexander M. Kulminski, Irina Culminskaya, Konstantin G. Arbeev, Liubov Arbeeva, Svetlana V. Ukraintseva, Eric Stallard, Deqing Wu, Anatoliy I. Yashin

**Affiliations:** Biodemography of Aging Research Unit, Social Science Research Institute, Duke University, Durham, NC, 27708–0408, United States of America; Medical University Hamburg, University Heart Center, GERMANY

## Abstract

Insights into genetic origin of diseases and related traits could substantially impact strategies for improving human health. The results of genome-wide association studies (GWAS) are often positioned as discoveries of unconditional risk alleles of complex health traits. We re-analyzed the associations of single nucleotide polymorphisms (SNPs) associated with total cholesterol (TC) in a large-scale GWAS meta-analysis. We focused on three generations of genotyped participants of the Framingham Heart Study (FHS). We show that the effects of all ten directly-genotyped SNPs were clustered in different FHS generations and/or birth cohorts in a sex-specific or sex-unspecific manner. The sample size and procedure-therapeutic issues play, at most, a minor role in this clustering. An important result was clustering of significant associations with the strongest effects in the youngest, or 3^rd^ Generation, cohort. These results imply that an assumption of unconditional connections of these SNPs with TC is generally implausible and that a demographic perspective can substantially improve GWAS efficiency. The analyses of genetic effects in age-matched samples suggest a role of environmental and age-related mechanisms in the associations of different SNPs with TC. Analysis of the literature supports systemic roles for genes for these SNPs beyond those related to lipid metabolism. Our analyses reveal strong antagonistic effects of rs2479409 (the *PCSK9* gene) that cautions strategies aimed at targeting this gene in the next generation of lipid drugs. Our results suggest that standard GWAS strategies need to be advanced in order to appropriately address the problem of genetic susceptibility to complex traits that is imperative for translation to health care.

## Introduction

Aging of populations in developed countries requires effective strategies to extend healthspan [1–3]. A promising solution could be to yield insights into genetic predisposition to diseases, their precursors (called endophenotypes [EP]), and mortality. Genome-wide association studies (GWAS) have been thought of as a major breakthrough in this endeavor.

The optimism is tempered, however, because studies using genome-wide resources face serious difficulties [4–6]. A fundamental source of difficulties in the genetics of complex health traits characteristic for modern societies is the lack of *direct* evolutionary selection against or in favor of such traits [7, 8]. This implies the lack of evolutionary established mechanisms for these traits. Accordingly, genes can be linked to complex traits through different mechanisms specific for a given period of life in a given environment. Then, the linkage between genes and these traits should be modulated by the individuals’ life course, i.e., by biodemographic processes [9].

Meanwhile, currently prevailing GWAS strategies rely heavily on collecting large samples disregarding the biodemographic aspect of the problem [4]. The basic hypothesis behind such a strategy is that if a part of phenotypic variance can be explained by genetic factors, then alleles with even small/moderate effects should gain statistical significance in large samples. Therefore, this logic assumes the existence of unconditional genetic risks. Conversely, it is argued that increasing the size of human disease cohorts merely increases the heterogeneity, making it even harder to detect risk alleles [6].

In this paper, we re-analyze the associations of SNPs discovered as correlates of lipids in a large-scale meta-analysis in [10]. The goal of our paper is to better understand the advantages and disadvantages of the traditional GWAS strategy in studying complex traits, i.e., when genetic effects are claimed to be weak. As an example, we use three successive generations participating in the Framingham Heart Study (FHS)—a study which was a part of meta-analysis in [10]. The paper considers all SNPs in detail which were directly genotyped in the FHS (i.e., they were present on the FHS genotyping array) and were associated with total cholesterol (TC) in [10].

## Results

The FHS includes participants from the FHS parental (FHS), Offspring (FHSO), and the 3^rd^ generation (3^rd^ Gen) cohorts (see Methods, “**Data**”). Basic characteristics of the study participants relevant to our analyses are given in Table 1.

**Table 1:** Basic characteristics of the genotyped samples of each sex.

Cohort	Sex	N	Birth cohorts, Mean (SD)	Age*, years, Mean (SD)	TC, mg/dL, Mean (SD)
**FHS**	M	613	1911 (6)	38.7 (6.5)	214.7 (38.8)
	W	916	1910 (7)	39.3 (7.0)	210.7 (42.4)
**FHSO**	M	1781	1937 (10)	36.0 (10.4)	198.5 (38.4)
	W	1969	1938 (10)	35.2 (9.9)	190.5 (37.7)
**3** ^**rd**^ **Gen**	M	1819	1963 (9)	40.3 (8.9)	192.8 (37.0)
	W	2069	1963 (9)	40.0 (8.8)	185.3 (33.7)

*Age is representatively given at baselines. TC is total cholesterol. SD is standard deviation

The FHS participants were of about the same age at baseline in each cohort. Women had lower TC levels than men. The TC levels tended to decline across the FHS generations. Because this secular trend can be attributed to improvements in medical care and prevention, we first investigated sensitivity of the associations of each of the ten selected SNPs (Table 2) with TC to individuals’ fasting status and lipid-lowering treatment (see Methods, “**Fasting versus random TC**” and “**Lipid-lowering therapy**”). The results show at most a minor role of these factors in these associations. Accordingly, the results are presented next regardless of the individuals’ procedure or treatment statuses.

**Table 2:** Characteristics of SNPs selected for the study.

N	SNP	Chr	Alleles, major/minor	MAF	Function	Gene	Trait^#^
**1**	**rs2479409**	1	A/G	0.34	near-gene-5	*PCSK9*	TC, LDL-C, metabolite levels, response to statin therapy, CHD, MI
**2**	**rs3177928**	6	G/A	0.14	UTR-3	*HLA-DRA*	TC, LDL-C, multiple sclerosis, Parkinson disease, ulcerative colitis, Hodgkin’s lymphoma, systemic sclerosis; rheumatoid arthritis
**3**	**rs1800562**	6	G/A	0.06	missense	*HFE*	Iron status biomarkers, hepsidin level, TC, LDL-C, transferrin glycosylation, hematology traits; red blood cell traits, hemoglobin, hematocrit, CVD risk factors
**4**	**rs9488822**	6	A/T	0.37	intron	*FRK*	TC, LDL-C, urate levels, AMD
**5**	**rs1564348**	6	T/C	0.16	intron	*SLC22A1*	TC, LDL-C, metabolic traits, prostate cancer
**6**	**rs10128711** *	11	C/T	0.23	intron	*SPTY2D1*	TC, AMD, urate levels
**7**	**rs11220462** **	11	G/A	0.13	intron	*ST3GAL4*	TC, LDL-C, liver enzyme levels
**8**	**rs3764261**	16	C/A	0.30	unknown	*CETP* ***	TC, LDL-C, HDL-C, tryglicerides, AMD, metabolic syndrome, lipoprotein-associated phospholipase A2 (Lp-PLA2) activity, hematological traits, waist circumference, CVD risk factors
**9**	**rs7206971**	17	G/A	0.49	intron	*EFCAB13*	TC, LDL-C
**10**	**rs1800961**	20	C/T	0.03	missense	*HNF4A*	TC, HDL-C, CRP, ulcerative colitis, type 2 diabetes

These ten SNPs were selected as described in Methods, “Selection of SNPs”

^*^ Lead SNP in [10] whereas the effect was reported for best SNP, rs10832963

** The association for total cholesterol in [10] is reported for rs11220463 which is tag SNP for rs11220462 available on the Affymetrix 500K array in FHS

***Closest gene

^#^ Associations of the selected SNPs and other variants from the same genes were assessed from GWAS reported at http://www.genome.gov/gwastudies.

MAF is minor allele frequency; Chr is chromosome; CRP is C-reactive protein, AMD is age related macular degeneration; TC is total cholesterol, HDL-C is high density lipoprotein cholesterol, LDL-C is low density lipoprotein cholesterol, CVD is cardiovascular disease, CHD is coronary heart disease, MI is myocardial infarction

### Associations of SNPs with TC in Nature meta-analysis and pooled FHS sample

First, we compared the associations for each SNP with TC from [10] with those observed in a pooled FHS sample. Results were shown in Table 3 (see columns “Beta*” and “Beta**” in this subsection). Given the effect sizes in [10], the most optimistic (i.e., minimal, when the FHS familial relatedness is disregarded) estimates of the sample sizes needed to achieve nominal significance (p = 0.05) meet (or nearly meet) the sample size in the pooled FHS sample for five SNPs (rs2479409, rs3177928, rs1564348, rs3764261, and rs1800961). For the other five SNPs, the estimates suggest substantially larger samples. Note that in case when familial relatedness is not disregarded, the estimates of the sample sizes are larger, which strengthens our results.

**Table 3:** Associations of SNPs with TC from the Nature meta-analysis and those evaluated in pooled FHS sample.

SNP	Nature meta-analysis			Pooled FHS sample						
	Beta* (SE)	P-value	N_p = 0.05_	N	Beta* (SE)	P-value	Beta** (SE)	P-value	Beta*** (SE)	P-value
**rs2479409**	1.96 (0.24)	3.8E-24	6,570	8,464	0.06 (0.57)	9.2E-1	0.04 (0.13)	7.3E-1	0.10 (0.11)	3.5E-01
**rs3177928**	2.31 (0.27)	4.0E-19	8,817	8,431	2.69 (0.82)	1.1E-3	0.62 (0.18)	6.4E-4	0.44 (0.16)	5.7E-03
**rs1800562**	-2.16 (0.43)	2.5E-08	21,532	8,501	-2.00 (1.17)	8.6E-2	-0.45 (0.26)	7.9E-2	-0.51 (0.23)	2.7E-02
**rs9488822**	-1.18 (0.20)	1.7E-10	17,456	8,387	-1.39 (0.57)	1.4E-2	-0.29 (0.12)	2.1E-2	-0.21 (0.11)	5.8E-02
**rs1564348**	2.18 (0.27)	9.7E-17	8,868	8,477	2.04 (0.76)	7.5E-3	0.43 (0.17)	1.1E-2	0.57 (0.15)	9.9E-05
**rs10128711**	-1.06 (0.22)	2.5E-08	28,474	8,428	-1.16 (0.65)	7.2E-2	-0.27 (0.14)	5.7E-2	-0.34 (0.13)	7.2E-03
**rs11220462**	2.01 (0.33)	2.1E-11	12,398	8,460	2.09 (0.81)	9.9E-3	0.46 (0.18)	9.7E-3	0.31 (0.16)	4.9E-02
**rs3764261**	1.67 (0.23)	6.7E-14	9,672	8,284	2.07 (0.61)	7.2E-4	0.45 (0.13)	8.8E-4	0.32 (0.12)	7.1E-03
**rs7206971**	1.01 (0.20)	1.1E-08	22,226	8,425	0.47 (0.55)	3.9E-1	0.13 (0.12)	2.8E-1	0.09 (0.11)	3.8E-01
**rs1800961**	-4.73 (0.66)	5.7E-13	8,700	8,484	-4.43 (1.59)	5.5E-3	-1.09 (0.35)	1.9E-3	-1.66 (0.31)	5.8E-08

^*^ The effect size beta is evaluated at baselines for directly measured TC in mg/dL to make comparison with the results of the Nature meta-analysis. N denotes sample size used in evaluation of beta at baselines in the FHS cohorts.

** The effect size beta is evaluated at baselines for 100×log_10_(TC).We use log-transformed TC in all further analyses because it corrects for deviation from normal distribution of the directly measured TC in the FHS.

^***^ The effect size beta (for 100×log_10_(TC)) and p-values are for the estimates of cumulative genetic effects over multiple FHS examinations.

N_p = 0.05_ denotes the estimates of the sample sizes which are needed to achieve nominal (p = 0.05) significance given the effect sizes observed in the Nature meta-analysis. These are the most optimistic estimates which disregard the FHS familial relatedness. The sample sizes become larger when familial relatedness is taken into account.

Table 3 shows, however, that p-values are better than expected for eight SNPs (those except rs2479409 and rs7206971) and worse than expected for one SNP (rs2479409). The worse estimate for rs2479409 is attributed to substantially weaker effect size in the FHS (Table 3, columns “Beta*”) than in [10] but not to insufficiency of the sample size. That is, having the effect size as in [10], the sample size in the FHS is sufficient to attain conventional significance p = 0.05 (Table 3, columns N_p = 0.05_ and N).

For four SNPs, for which the estimates suggested larger samples than in the FHS (rs1800562, rs9488822, rs10128711, and rs11220462), the effect sizes were virtually the same as in the Nature meta-analysis. Despite suggested insufficiency of the FHS sample for given effects, these SNPs showed either nominal or suggestive effect (p<0.1) associations.

The directions of the effects in the pooled FHS sample were concordant with those in [10] for all SNPs.

### Cumulative associations of SNPs with TC over the life course of FHS participants

The cumulative estimates (see Methods, “**Analysis**”) improve significance for half of SNPs (rs2479409, rs1800562, rs1564348, rs10128711, and rs1800961) by increasing the effect sizes and decreasing standard errors (Table 3, columns “Beta**” and “Beta***”). This improvement can be dramatic. For example, the largest improvement of five orders of magnitude was observed for rs1800961 when p = 5.8×10^−8^ virtually reached genome-wide significance level (p_GW_ = 5.0×10^−8^) in the same sample of 8,484 individuals.

For the other five SNPs (rs3177928, rs9488822, rs11220462, rs3764261, and rs7206971), the cumulative estimates suggested that the baseline-based associations were an overestimation.

### Associations of SNPs with TC across sexes and generations

The above results (i.e., weaker effects in the FHS (rs2479409 and rs7206971) than in the Nature meta-analysis and disagreement between the expected and observed p-values in the FHS) suggest that better understanding the architecture of genetic associations can be crucial for improving efficiency of GWAS of inherently heterogeneous traits for which unconditional associations with genetic factors can be problematic.

**Fig 1:**
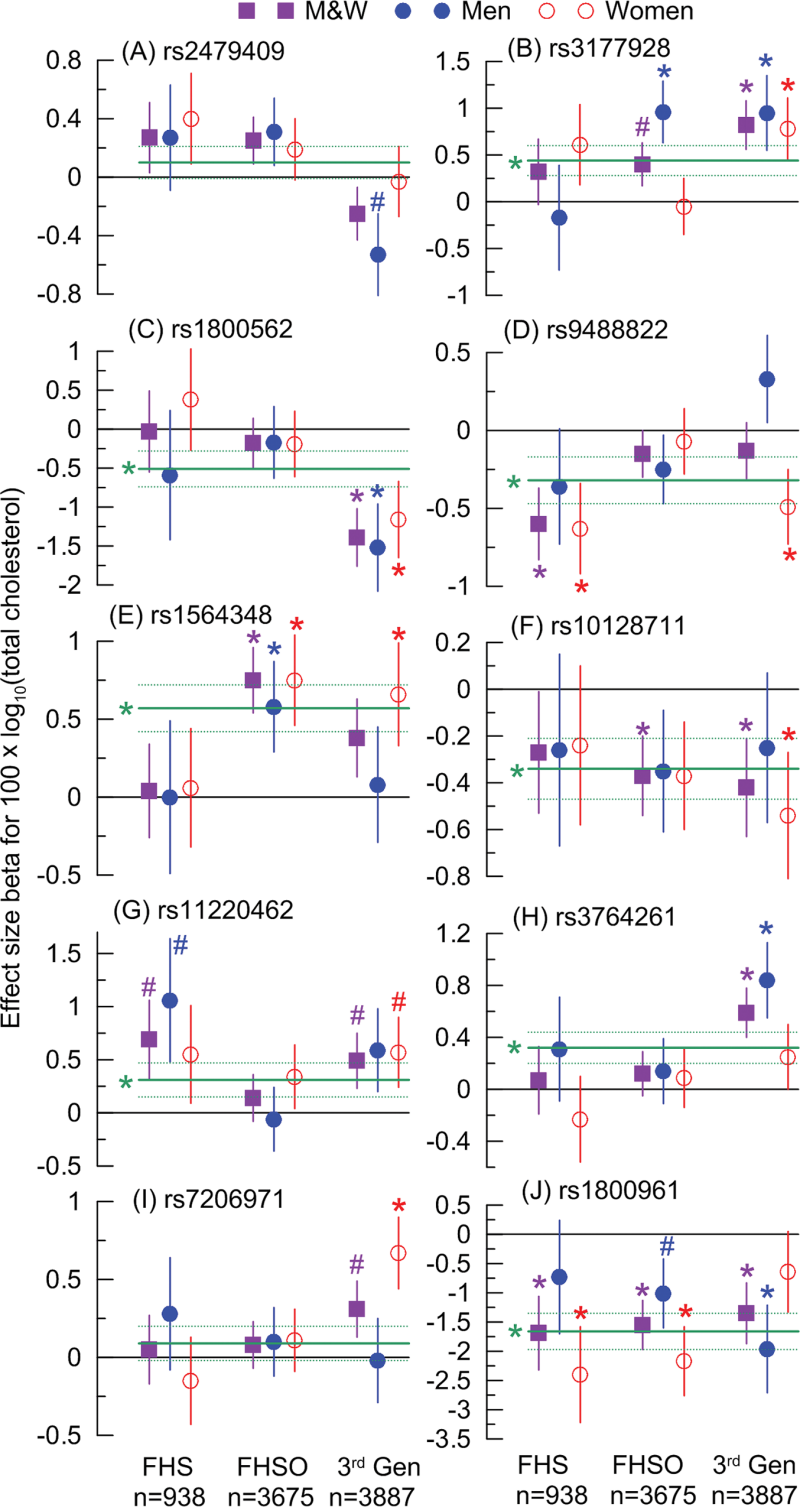
Sex-specific associations of SNPs with TC across three FHS generations. Symbols show the effect size beta for the minor allele. Solid horizontal green line depicts the effect size beta in the pooled FHS sample. Bars and horizontal dotted lines show standard errors. An asterisk and a number symbol show significant (p≤0.05) and suggestive-effect (0.05<p≤0.1) associations, respectively. N in the lower inset denotes maximal number of individuals used in the analyses in each cohort at baseline. Numerical estimates are given in S2 Table.

Here we examine the role of FHS generations, as reliable proxies of environmental exposures, in genetic associations. Results were shown in Fig 1. It is seen that the effect sizes are consistent across sexes and generations only for one of ten SNPs (rs10128711). This is what is expected in case of unconditional connections of SNPs with traits. The other nine SNPs have, however, markedly different effect sizes in these subgroups. Importantly, the effect sizes are markedly different even if the FHS original cohort is disregarded, i.e., in the FHSO and 3^rd^ Gen, which are the largest cohorts of virtually the same sample sizes. This inconsistency is also supported by the analyses of 19 proxy SNPs (S1 Table).

Fig 1 helps clarify that small effect sizes of rs2479409 and rs7206971 in the pooled FHS sample (Table 3) reflect superposition of complex modes of their action rather than their true weak effects. Indeed, Fig 1A shows that the negligible effect of rs2479409 in the pooled FHS sample is the result of superposition of antagonistic effects in different FHS generations. Weak effect of rs7206971 in the pooled sample (Fig 1I) reflects its sex and generation specifics.

Fig 1 shows 16 significant associations in independent demographic cohorts of men and women from different generations. By contrast, only three significant associations are expected by chance for these 60 tests (i.e., three cohorts of men and women for 10 SNPs). This implies 5.3-fold highly significant (p = 6.3×10^−5^) excess of the observed associations compared to those by chance.

The results in Fig 1 reveal an increase in the number of significant associations across generations in a sex-specific manner (emphasized in S1 Fig). Fig 1 shows suggestive-effect or nominal significance for seven SNPs (rs2479409, rs3177928, rs1800562, rs9488822, rs11220462, rs3764261, and rs7206971) in the samples of men (Fig 1A and 1H), women (Fig 1B, 1D, 1G and 1I), or both sexes (Fig 1C) in the 3^rd^ Gen cohort but not in the FHSO. Importantly, clustering of associations for these seven SNPs in the 3^rd^ Gen cohort is attributed to larger effect sizes in the youngest FHS generation and is not the result of the sample size differences between these cohorts because they are virtually of the same size. In contrast, the reverse situation with significant associations in the FHSO but not in the 3^rd^ Gen is observed only for two SNPs (rs1564348 for men and rs1800961 for women). This disproportion (i.e., seven vs. two SNPs) in samples of the same size supports the trend on clustering of genetic effects in the youngest FHS generation.

### Potential for improving GWAS efficiency

If clustering of significant associations in specific demographic cohorts is real, then just increasing sample size by pooling data from all FHS cohorts and disregarding demographic specifics (i.e., following the traditional GWAS strategy) may heavily under-use available resources. Besides, because this strategy averages the effects across different demographic groups, it may provide misleading results in terms of clinical translation.

**Fig 2:**
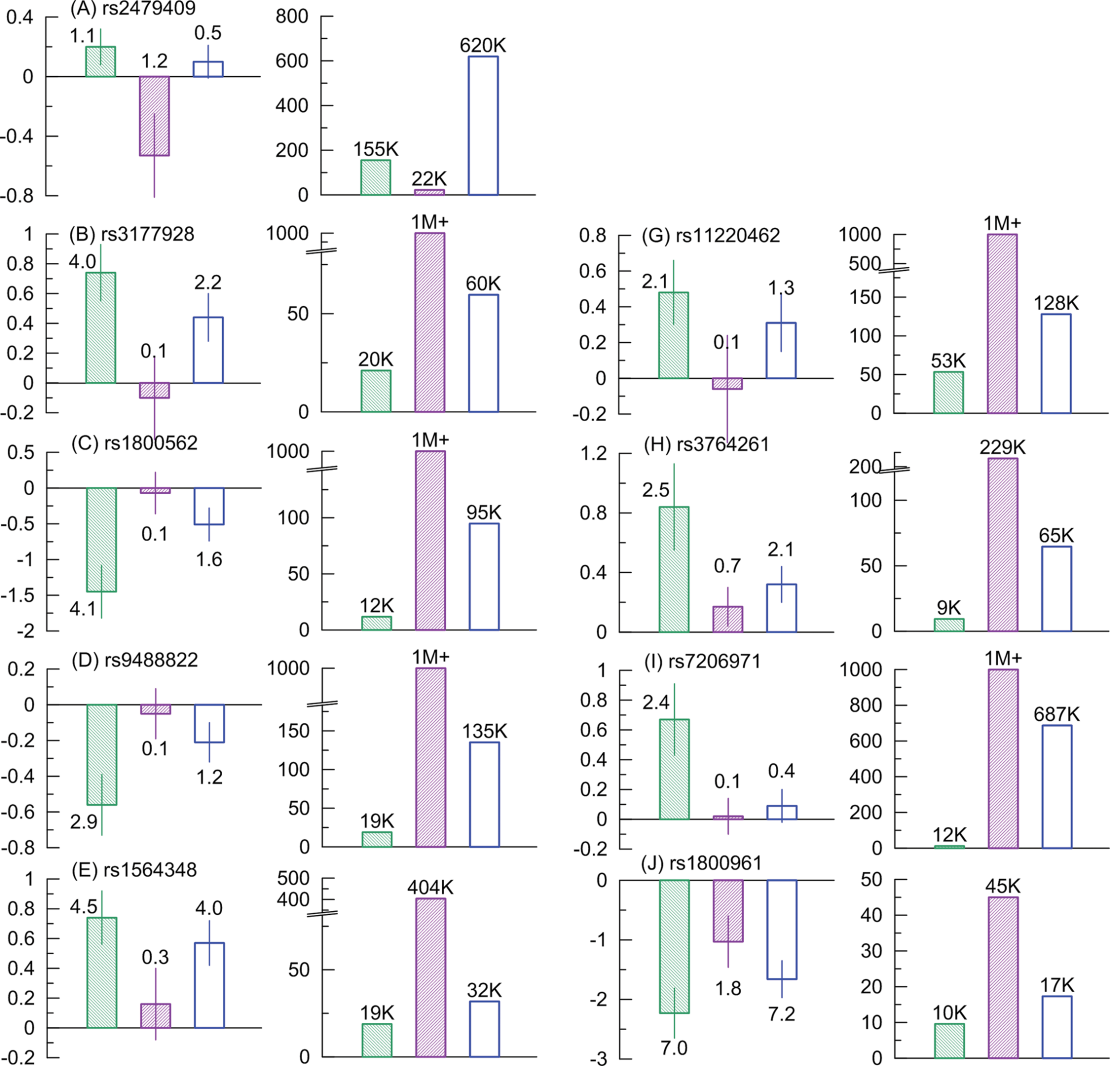
Associations of SNPs with TC in more homogeneous subsamples of FHS participants. Columns with labels and SNP IDs (labeled columns) show the effect sizes (bars) and -log_10_(p-values) (numbers) in the S-subsample (green color), W-subsample (purple color), and the entire FHS sample (blue color). The level of nominal significance is -log_10_(0.05) = 1.3. The S- and W-subsamples are defined in the “**Potential for improving GWAS efficiency**” section. Thin bars show standard errors. Unlabeled columns show the estimates of the sample sizes (shown by bars and by numbers in thousands (K) and millions (M)) needed to achieve genome wide significance (p = 5×10^−8^) based on the estimates of the effect sizes in the labeled columns. Colors in the unlabeled columns correspond to colors in the labeled columns. Note breaks of the y-axes that was done for better resolution of smaller sample sizes. Numerical estimates are given in S3 Table.

Specifically, Fig 2 (labeled columns) shows the effect sizes for each of the nine SNPs (with inconsistent effects, i.e., except rs10128711) in the entire FHS sample and in two subsamples. One subsample (S-subsample) includes demographic cohorts, which have homogeneous effects when heterogeneity coefficient (I^2^, see Methods, “**Analysis**”) is zero for the effects between cohorts included in the S-subsample; see more details in S3 Table). The S-subsample provides the strongest support for the results of the meta-analysis in [10]. The other subsample (W-subsample) was selected in the same manner but it provides the weakest support to the results in [10]. This strategy minimizes heterogeneity in the effects within the S- and W-subsamples and maximizes heterogeneity between them by taking into account demographic specificity (S3 Table).

The effect sizes in the S- and W-subsamples are markedly different with high heterogeneity ranging from I^2^ = 58.0% (rs11220462) to I^2^ = 88.4% (rs1800562). When this demographically-driven heterogeneity is disregarded, the effect size in the entire FHS sample is an average of the effects in both subsamples (Fig 2, labeled columns, blue bars).

Disregarding this high heterogeneity may not be plausible as also supported by highly significant enrichment of the significant associations (see above). Then, what are the benefits if this heterogeneity is not disregarded? We have evaluated the sample sizes, which are needed to achieve p_GW_ = 5×10^−8^ based on the effect sizes in each sample shown in Fig 2 (labeled columns). Importantly, the effect sizes and sample sizes are two critical parameters, which are of primary interest for clinical translation (effect size) and GWAS efficiency (sample size).

Fig 2 (non-labeled columns) shows that, for all SNPs, the sample sizes needed to achieve p_GW_ are smaller for the S-subsample than for the entire sample by a factor ranging from 1.7 (rs1564348) to 55.5 (rs7206971). Contrarily, for all SNPs (except rs2479409), the sample sizes needed to achieve p_GW_ are substantially larger for the W-subsample than for the entire sample by a factor ranging from 1.5 (rs7206971) to at least 16.8 (rs3177928). For five SNPs in the W-subsample, the effects sizes are negligible because they require samples with more than 1,000,000 people to gain significance. S3 Table ensures that differences between the effect sizes in the S- and W-subsamples are not explained by the sample size differences because there is no direct correlation between the effect sizes and the sample sizes in these subsamples. Although for rs2479409 the size of the W-subsample is smaller than the size of the S-subsample, the W-subsample shows an opposite effect to that reported in [10].

### The role of age and birth cohorts in the associations of SNPs with TC

Because individuals in the FHS cohorts were of different age at biospecimens collection, sensitivity of the effects to generations (Fig 1) may be due to environmental exposures and/or age-related processes. To gain some insights into the role of these mechanisms in the observed associations, the analyses in this subsection were conducted in stratified samples of the FHSO and 3^rd^ Gen cohorts, appropriately matched by ages at biospecimens collection and ages at measurements of TC (see Methods, “**Bio-demographic processes and genetic effects**”). The goal of these analyses was two-fold. First, given clustering of significant and suggestive effect associations with strongest effects in the 3^rd^ Gen cohort (12 of 20 associations, see Fig 1), we examined potential sensitivity of these associations to birth cohorts. Second, we examined age-overlapping sub-cohorts from the FHSO and 3^rd^ Gen cohorts in an attempt to delineate the predominant influence of age or birth cohorts in these associations. Given this two-fold goal, each of the FHSO and 3^rd^ Gen cohorts was stratified into two sub-cohorts of younger and older ages. To minimize the sample-size-related biases in each cohort, we used median ages of 40 (3^rd^ Gen) and 60 (FHSO) years (see S2 Fig) as cutoffs for this stratification.

**Fig 3:**
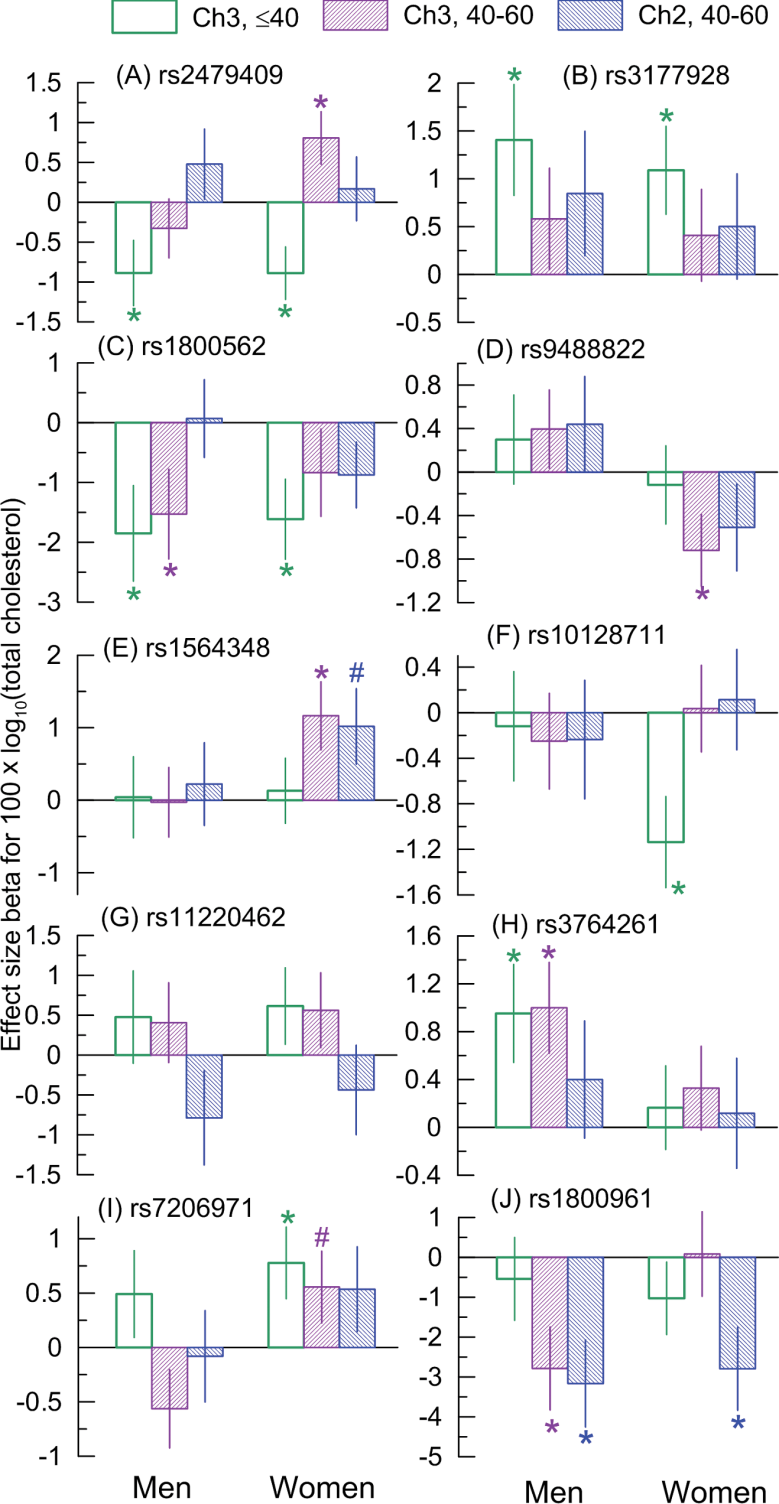
Birth-cohort-specific associations of SNPs with TC for men and women in FHSO (Ch2) and 3^rd^ Gen (Ch3). The samples were stratified into younger and older sub-cohorts as defined by median cut-off for the age at biospecimens collection, i.e., 40 years in the 3^rd^ Gen and 60 years in the FHSO (see upper inset). There were virtually no individuals aged older than 60 years in the 3^rd^ Gen and younger than 40 years in FHSO (see S2 Fig). Wide bars show the effect size beta. Thin bars show standard errors. An asterisk and a number symbol show significant (p≤0.05) and suggestive-effect (0.05<p≤0.1) associations, respectively. Numerical estimates are given in S7 Table.

Five of 12 associations in the 3^rd^ Gen cohort are primarily explained by dominant and significant effects in the younger sub-cohort (Fig 3A men, 3B men, 3B women, 3C women and 3F women). The effect sizes in the older 3^rd^ Gen sub-cohort were substantially smaller, which explained the lack of their significance. An important result was that weak effects in this older 3^rd^ Gen sub-cohort were resembled in the age-matched FHSO sub-cohort.

For three more associations, we **observed** clustering of the effects in the older 3^rd^ Gen sub-cohort (Fig 3D women, 3E women and 3J men). Again, as in the case of the weak effects discussed above, strong effects were also resembled in the age-matched FHSO sub-cohorts.

Thus, eight of 12 significant associations in the 3^rd^ Gen cohort were sensitive to birth cohorts. Replications of the lack of the effects (Fig 3A men, 3B men, 3B women, 3C women and 3F women) and of the presence of the effects (Fig 3D women, 3E women and 3J men) for these eight associations in the age-matched FHSO sub-cohort suggest a stronger role of age-related processes in the observed sensitivity rather than the role of the environment.

The remaining four of 12 associations were not sensitive to birth cohorts in the 3^rd^ Gen (Fig 3C men, 3G women, 3H men and 3I women). The effect was resembled in the age-matched FHSO sub-cohort for only one SNP (Fig 3I women). Weak effects for the other three associations in the age-matched FHSO sub-cohort, and strong effects in the 3^rd^ Gen cohort, indicate a predominant role of the environment (characterized by generations) over the age-related processes.

Fig 3 provides two more important insights as discussed next.

#### Antagonistic effects of rs2479409

**Fig 4:**
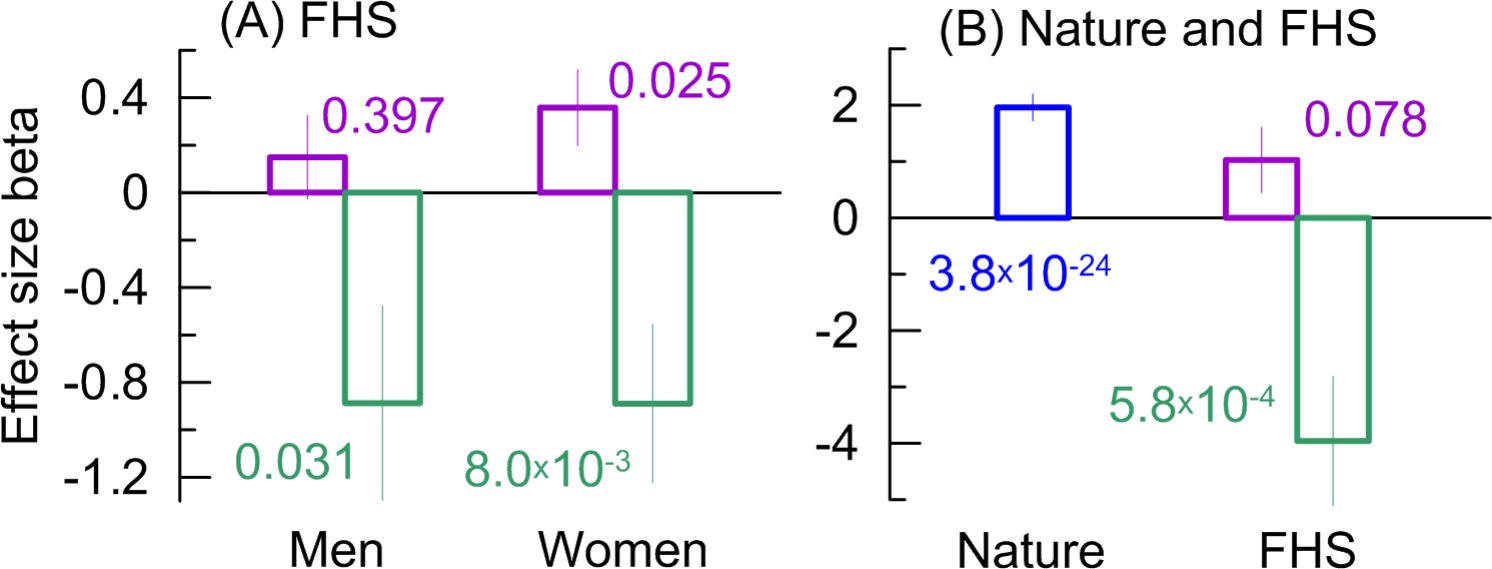
Antagonistic effects of rs2479409. (A) The antagonistic effects in the samples of the FHS men and women. (B) The effects in the Nature meta-analysis (Nature) and the pooled sample of the FHS men and women (FHS). Purple color denotes detrimental effects in the pooled samples from the FHS original cohort, the FHSO cohort, and the older sub-cohort (defined in Fig 3) of the 3^rd^ Gen cohort. Green color denotes protective effects in the younger sub-cohort (defined in Fig 3) of the 3^rd^ Gen cohort. Numbers show p-values. Wide bars show the effect size beta. Thin bars show standard errors. Numerical estimates are given in S8 Table.

The analyses support antagonistic effects of rs2479409 in younger and older 3^rd^ Gen women (Fig 3A). This antagonism explains the negligible effect of rs2479409 in the entire sample of 3^rd^ Gen women (Fig 1A). This women-specific antagonism was supported by the men-specific antagonism in the effect of rs2479409 across generations (Fig 1A), which explained negligible effect of rs2479409 in the entire FHS sample (Fig 2A). Importantly, the protective effect of the minor allele for 3^rd^ Gen men (Fig 1A) attains better significance and larger effect size in the younger sub-cohort. As a result, the antagonistic effects become more pronounced in each sex with the consistent significant protective role of rs2479409 in younger men (beta = -0.89, p = 3.1×10^−2^) and women (beta = -0.89, p = 8.0×10^−3^) (Fig 4A). This protective effect becomes highly significant in these samples combined (beta = -0.88, p = 8.4×10^−4^). Re-evaluating this pooled effect in the same TC units as in the Nature meta-analysis (i.e., for untransformed TC measured in mg/dL), we determined that the size of the protective effect (beta = -3.96, p = 5.8×10^−4^) was two-fold larger than that of the detrimental effect in the Nature meta-analysis, making the antagonistic effects to be remarkably strong (Fig 4B).

#### Clustering of the effect of rs10128711

Fig 1F supports a homogeneous contribution of one of the 10 SNPs, rs10128711, into association with TC across FHS generations. However, Fig 3F shows that the effect of this SNP is attributed to younger women whereas its effects are lacking in older women. Replication of the lack of the effects in two independent sub-cohorts of women of the same older age (40 to 60 years) from different generations implies that a contribution of rs10128711 into association with TC across FHS generations is also non-homogeneous. Combining this result with those in Fig 1 for the other nine SNPs, our analyses do not support unconditional associations of SNPs available in the FHS with TC.

### Biological role of genes

Ten SNPs selected for detailed analyses were located within or near the protein-coding genes (Table 2). Previous findings in GWAS (http://www.genome.gov/gwastudies) for these 10 SNPs and for other variants from the same genes are detailed in Table 2. Next, we emphasize the similarity in functions of these genes, which may be involved in a system of interlinked processes beyond lipid metabolism.

#### Genes with known functions

Transcription factor *HNF4A* (rs1800961) plays a crucial role in hepatocyte differentiation and the maintenance of liver function [11]. *HNF4A* regulates the expression of the wide diversity of genes [12] including a serine protease *PCSK9* (*r*s2479409) and organic cation transporter (*SLC22A1*, rs1564348). Hepatic *PCSK9* that regulates cholesterol homeostasis through the LDL receptor, may have a role in liver regeneration [13] and may link lipid and thyroid metabolisms [14]. Importantly, plasma *PCSK9* levels may correlate with age in a sex-specific manner [15] and can significantly differ in pre- and post-menopausal women [16], which may pinpoint a biological mechanism of the observed antagonism for rs2479409. Cross-talk of *HNF4A* with *SLC22A1* is in line with functional interactions between cholesterol homeostasis and drug metabolism [17].

The hemochromatosis (*HFE)* (rs1800562) gene controls iron absorption [18]. Iron overload in the liver contributes to lipid peroxidation and DNA damage [19]. In addition, the *HFE* gene has immunomodulatory functions [20]. In line, studies show that *HNF4A* plays a role in iron metabolism [21], and promotes immune regulatory genes [22].

*HLA-DRA* (rs3177928) is related to MHC class II genes that play a central role in the immune system and have been associated with the suppressive effects of iron-binding proteins [23]. Also, *HLA-DR* has the potential procoagulant activity [24].

*FRK* (rs9488822) encodes protein with a potential role in cell cycle regulation and growth-inhibitory effects. Mice deficient in the *FRK* had decreased the circulating levels of thyroid hormone T3 associated with iron deficiency [25]. T3 regulates lipid metabolism and promotes hepatocyte proliferation and liver regeneration [25, 26].

The rate of liver's regeneration declines in aging organisms and there is evidence for the crucial role of epigenetic silencing in the age-dependent inhibition of liver proliferation [27]. *HNF4A* plays an important role in the establishment of epigenetic modifications in hepatocytes [28]. Histone-binding protein, Spt2 (*SPTY2D1*, rs10128711) plays a role in rDNA transcription through chromatin remodeling [29] and may function as cofactor of HNF4A in epigenetic control [30].

These seven genes may pinpoint a system of interlinked processes with potentially a key role of missense variants (rs1800961, rs1800562) in this functional crosstalk. Beside of role in lipid homeostasis, their connection with age-related processes may be: (i) through dysregulation of iron homeostasis with age, which results in an increase of iron in a sex-specific manner [31], (ii) through age-related degradation of the immune system [32] and hormonal changes [33], and (iii) through decline in stress resistance, accumulation of DNA damages, and decrease in regenerative capacity of the liver with age [34].

*ST3GAL4* (rs11220462) is important in the maintenance of hemostasis [35] and may play a role in mediating proatherogenic events [36]. The CETP gene (rs3764261) has been connected with HDL levels. Also, CETP plasma levels are thought to be associated with blood coagulability [37]. Thus, these two genes may represent components of the age-specific regulatory network in hemostasis. Given also antithrombotic actions of HDL [38], a link of higher HDL levels with healthy aging and longevity [39], and a state of hypercoagulability of centenarians [40], it is possible that these genes may be involved in buffering mechanisms in exceptional longevity [41].

#### Function of EFCAB13 (rs7206971)

Function of *EFCAB13* gene remains unknown. However, the possible relation of *EFCAB13* to thyroid hormone T3 [42] suggests a systemic link with the other seven genes discussed above. Accordingly, potential age-specifics suggested in Fig 3I for men may be related to hormone balancing.

## Discussions

GWAS often rely on “the benefits of the large sample sizes achievable through collaboration” [43] for detecting risk alleles of complex traits. Such strategy assumes the existence of unconditional genetic components in health risks that is, generally, problematic (see below). As a consequence, this strategy disregards the possible complexity of genetic effects on complex traits that decreases GWAS efficiency [6]. To better understand the advantages and disadvantages of the traditional GWAS strategy in the analysis of complex traits, we re-examined in detail the associations of SNPs associated with TC in a Nature meta-analysis [10]. For our detailed analysis we selected all ten SNPs reported in [10] which were directly genotyped in three generations of humans participating in the FHS—a study which was a part of that meta-analysis. A specific overarching goal of the analyses was to better understand whether or not these SNPs can be considered as unconditional risk factors for complex traits such as TC. Below, we discuss sensitivity of the associations of SNP with TC to demographic factors such as birth cohort, sex, and age. We also provide a potential explanation of this sensitivity for complex traits such as TC.

### Evidence of complex role of genes in susceptibility to TC

Weaker effects of two SNPs (rs2479409 and rs7206971) in the pooled FHS sample than in [6] and disagreement between the expected and observed p-values for most SNPs in the FHS (Table 3) suggest a role of heterogeneity in these associations.

More detailed analyses showed inconsistent effects of nine of ten SNPs across the different FHS generations in a sex-specific or sex-unspecific manner (Fig 1). A potential explanation for this is given in the next subsection. The highly significant 5.3-fold excess (p = 6.3×10^−5^) of significant associations in these demographic groups, compared to those expected by chance, suggests that the observed enrichment is likely real. The probability of false findings is also reduced by the use of repeated measurements in the analyses, which improves the robustness of the estimates (see Methods, “**Analysis**”).

An important result was the clustering of significant associations with the strongest effects in the youngest 3^rd^ generation FHS cohort. This clustering is not explained by sample size, potential differences in the procedures of TC measurement in the FHS, or the role of lipid-lowering therapy. These results show that demographic factors can, in part, explain the lack of replication of genetic effects in studies focusing on different demographic groups.

Pooling demographic groups with homogeneous effects, which provide the strongest support to the results in [10], and groups with the weakest support highlights high heterogeneity between these two samples, which ranges between I^2^ = 58.0% and I^2^ = 88.4% for different SNPs. Unfolding this demographic heterogeneity has substantial potential for improving GWAS efficiency (Fig 2).

To gain some insights on mechanisms connecting SNPs with TC [7, 44], we examined the role of age and birth cohorts, which are considered as proxy of the age-related processes and the environmental exposures. These analyses suggest that the age-related and environmental mechanisms are likely associated with different groups of SNPs. Although these results should be interpreted with caution given the relatively small samples, they clearly indicate that much more rigorous analyses beyond the traditional GWAS are needed to improve our understanding of the genetic influence on complex traits.

The analyses highlight the consistent antagonistic effects of rs2479409, which are seen across generations and birth cohorts. Unlike the pure detrimental role of rs2479409 suggested in [10], our analyses suggest that its role may change from detrimental to protective. Strong and highly significant protective effect of this SNP is limited to the youngest men and women of the youngest FHS generation (Fig 4). The change from the detrimental effect to the protective effect may be due to age-related mechanisms (as suggested by the biological function of *PCSK9*) and/or environmental changes characteristic for upcoming generations. This is an important result, which requires thorough attention because the gene for this SNP (*PCSK9*) is a target of a new class of cholesterol lowering drugs, which inhibit its expression [45, 46].

The analyses of the role of age and birth cohorts do not support homogeneous effect of the tenth SNPs (rs10128711) either. Thus, our analyses do not support unconditional associations of each of the ten directly genotyped SNPs with TC. Accordingly, assuming unconditional connections of SNPs with lipids would be less plausible than assuming complex role of these SNPs in lipid metabolism.

Analyses of the biological role of genes for these SNPs show that these genes are likely to be in cross-talk and are involved in different systems of interlinked processes related to aging.

### Empirical basis underlying complexity of gene actions on age-related traits

Men and women have different life expectancies and different sensitivities to factors causing diseases [47–49]. As long as it is believed that these traits can have genetic origins, sexual dimorphism in phenotypic expression will eventually highlight differences in the genetic regulation of these traits in men and women. The genetics of sexual dimorphism are highlighted by the X and Y chromosomes [50], and by differences in the functioning of genes at loci on autosomes (beyond linkage with the X and Y chromosomes) which can be attributed to different physiology (e.g., hormonal and insulin regulation [51]) and/or to different behavioral and psycho-social attitudes as modulators of genetic effects [49].

The evolutionary constraints do not support the existence of unconditional genetic components in age-related traits [7]. Lipid-related genes represent a vivid example. Indeed, because lipids are involved in energy metabolism and are a part of cellular components, the lipid genes are directly associated with diet and lifestyle [47, 52, 53]. These were the exposures which experienced dramatic changes during recent centuries [8, 54–56]. This change implies that evolutionarily-selected genetic mechanisms of even normal lipid metabolism are under exposures which differ from those in the past, whereas the evolutionary constraints do not support direct selection of genes against or in favor of abnormal EPs causing age-related diseases.

Conceptually, the sensitivity of genetic effects to age-related processes stems from changes occurring in a human organism over the life course. For example, biodemography, gerontology, and epidemiology provide firm evidence on the variation of various health traits over the organism’s life. These studies, for example, emphasize changes in various EPs over the life course such as the levels of physiological biomarkers [57–59]. The age-related processes are also manifested in changes of the risks of diseases [60] or death with age. Again, as long as these risks have a genetic basis, the observed changes in phenotypic expression over the life course imply sensitivity of genetic effects to age-related processes. Candidate-gene studies support this conclusion by showing differential roles of genes in complex traits at different periods of human life [61–66]. A specific case of the age-related sensitivity of genetic effects is implicated in the widely known hypothesis of antagonistic pleiotropy [67].

### Implications

Our results lead to five implications. First, they show that the mechanistic concept of replication of association at the same variant, with the same allele, in the same direction with the same trait in different demographic groups may be problematic that supports concerns on replication emphasized elsewhere [7, 68–71]. Second, they explicitly illustrate that “increasing the size of human disease cohorts is likely only to scale the heterogeneity in parallel” [6], with elusive chances for success. That is, just increasing the sample size without unfolding heterogeneity of complex traits such as TC may substantially under-use resources available in GWAS. The same conclusion is likely to be valid for the meta-analyses of the results of different studies. Third, they show that better understanding of the phenotype architecture [5], given demographic context [4], is crucial for characterizing the genetic influence on complex traits. Fourth, age-related processes in each sex in a specific environment may be essential factors in genetic susceptibility to complex traits. Fifth, using longitudinally measured information on an EP can substantially benefit GWAS.

Taken together, our results and their implications suggest that standard GWAS strategies need to be advanced in order to appropriately address the problem of genetic susceptibility to complex traits. This is crucial not only for a better understanding of the genetic origin of traits, but also for efficient use of genetic discoveries in health care.

## Methods

### Data

The FHS (N = 14,428 participants) design has been previously described [72–74]. Briefly, the FHS original cohort was launched in 1948 and included N = 5,209 respondents aged 28–62 years at baseline. These individuals have been biennially examined over nearly 60 years. The FHS Offspring (FHSO) cohort was launched 22 years later and included respondents (N = 5,124) aged 5–70 years at baseline who were mostly biological descendants and their spouses of the FHS participants. The FHSO respondents have been examined approximately every four years. The 3^rd^ Generation (3^rd^ Gen) cohort was launched in 2001 and consisted primarily of the biological descendants (N = 4,095) of the FHSO participants (one baseline examination is available via dbGaP). Measurements of total cholesterol are available at multiple examinations in the FHS/FHSO and one measurement is available in the 3^rd^ Gen cohort. Biospecimens were mostly collected in the late 1980s and through the 1990s from surviving participants [75, 76]. Genotyping of 9,167 FHS participants was conducted using Affymetrix 500K array [74].

### Selection of SNPs

In total, we have identified 143 SNPs associated with lipids in meta-analysis in [10]. Of these SNPs, 25 were on the Affymetrix 500K array in the FHS, a study which was included in that meta-analysis. Only 10 of them were, however, associated with total cholesterol (TC) in [10]. TC was chosen because it was measured at the majority of examinations in the FHS original cohort and at all examinations in the FHSO. These 10 directly genotyped SNPs, which were used in meta-analysis in [10], were the major focus of our analyses (Table 2).

### Analysis

Following [10], associations of the selected SNPs with TC (mg/dL) were characterized using an additive genetic model with the minor allele considered as an effect allele. The models were fitted using a mixed effects regression (SAS; release 9.3, Cary, North Carolina, USA).

The TC in the FHS original and FHSO cohorts was measured on multiple occasions during follow-up of the same individuals. We used two strategies in estimating the associations. First, following common practice, we used baseline information on TC. To minimize potential bias associated with within-family correlation, a two-level model was fitted. These analyses, however, disregard rich information on repeated measurements of TC.

Because the latter information may add power to the analyses [77], we used the second strategy which evaluated the effects given repeated measurements, i.e., it evaluated the associations for SNPs given measurements of TC for individuals of a given age at each selected examination. This was the main strategy in our analyses. We used TC measurements at: (i) 16 examinations in the FHS original cohort covering the entire range of follow-up, and (ii) all seven examinations in the FHSO cohort. This strategy uses a three-level mixed effects regression model to account for familial and repeated-measurements correlations. Because information on TC in the 3^rd^ Gen cohort is available only at baseline, there is no difference between the two adopted strategies in evaluating the effects in this cohort.

Importantly, the second strategy can substantially improve reliability of the estimates because it evaluates the cumulative genetic effects over follow-up. The results become more reliable because of offsetting of potential problems associated with: (i) possible inconsistencies in measurements of TC across examinations (regardless of the nature of those inconsistencies), and (ii) missing TC measurements at some examinations. Substantially, these estimates represent cumulative genetic effects over a portion of the individuals’ life course.

A two-level mixed effects regression model was also used to evaluate the associations at specific examinations for the analyses of the role of fasting (see below) and lipid lowering therapy (see below), as well as for the analyses of age- and birth-cohort-specific effects (see below).

To account for deviation of the TC frequency distributions from normality, we used log-base-10-transformed values (multiplied by 100 for better resolution).

The models were adjusted for: (i) cross-sectional age, (ii) whether the DNA samples had been subject to whole-genome amplification [78], (iii) sex, and (iv) FHS cohort differences.

The sample sizes were estimated using Quanto (http://biostats.usc.edu/). We assumed the same additive genetic model, which was used in the association analyses. We used population mean and standard deviation (SD) for TC = 194 (SD = 38) mg/dL observed in the FHS.

Heterogeneity coefficient I^2^ [79] was evaluated using *metafor* package in R. The I^2^ coefficient can be interpreted as the percentage of the total variability in a set of the effect sizes due to true heterogeneity, i.e., due to between-sample variability.

### Fasting versus random TC

In early examinations of the FHS original cohort, the study measured TC levels using a sample of random (i.e., casual) whole blood, whereas in the FHSO and 3^rd^ Gen cohorts most levels were measured after a fast. Increasing evidence, including large-scale studies, suggests that fasting may not substantially change levels of lipids [80]. Additionally, blood in the FHS was drawn at the end of long (lasting several hours) examinations, which diminished potential differences between the random and fasting levels [81].

In order to verify whether or not this is indeed the case for TC in FHS, we selected examinations with known fasting status and evaluated the association of TC with fasting. Fasting was dichotomized as fasting 12+ hours for fast and unknown fasting status or less than 12 hours of fasting otherwise. S4 Table shows that most individuals in the FHS original cohort had not being intentionally fasting, whereas most individuals in the FHSO and 3^rd^ Gen cohorts had. Despite this procedural difference, no significant differences in TC levels are seen at eight of 12 examinations in all cohorts.

Significant associations are observed at four examinations, i.e., at 8^th^ and 9^th^ (FHS), 7^th^ (FHSO), and 3^rd^ Gen. The differences in fasting and random TC levels were, however, either very minor (7^th^ FHSO examination and 3^rd^ Gen) or they were observed in samples with small number of fasting individuals (6.9% and 4.3% at 8^th^ and 9^th^ FHS examinations). Accordingly, for consistency with other examinations, these minor and potential differences in fasting and random TC levels at four of 12 examinations were disregarded and all individuals (i.e., regardless of their fasting status) were included in the analyses.

### Lipid-lowering therapy

We observe the same or significantly *higher* TC levels at 12 (of 15) examinations in participants of the FHS original (7 of 8) and FHSO (5 of 7) cohorts who were on lipid-lowering therapy compared to the others (S5 Table). These results were consistent at early visits in these cohorts. At later visits in FHS/FHSO and at the visit in 3^rd^ Gen, significantly lower TC levels were observed in individuals who were on lipid-lowering therapy compared to the others.

Because the FHS original and FHSO cohorts were launched so long ago (see Methods, “**Data**”), the number of individuals who were on lipid-lowering therapy in early examinations in these cohorts was small (S5 Table). This suggests that lipid-lowering treatment at early visits was likely administered to individuals with very high lipid levels, which likely remained higher in these individuals even after treatment, compared to those who were not on lipid-lowering therapy. Accordingly, we believe that it would be inappropriate to exclude such individuals from the analyses. Therefore, all individuals at early visits in FHS and FHSO were considered.

The finding of lower TC levels in individuals who were on lipid-lowering therapy at later examinations in FHS/FHSO and in 3^rd^ Gen cohorts might suggest that such individuals should be excluded from the analyses in order not to dilute the associations. However, it is actually unknown whether or not associations of the selected SNPs with TC are indeed affected by lipid-lowering therapy.

To clarify this question, S6 Table shows the associations of each selected SNP with TC measured at 6^th^ and 7^th^ visits in FHSO and at baseline in the 3^rd^ Gen (at which lipid-lowering therapy was associated with lower TC levels) for all individuals, and for those who had been on lipid-lowering therapy excluded. S6 Table suggests that, in general, excluding individuals treated by lipid-lowering medications a priori is not warranted because in many cases the associations are either the same or better when such individuals are retained in the analyses.

Given at most a minor role of lipid-lowering therapy for the selected SNPs, all individuals (i.e., regardless of their treatment status) were included in the analyses.

### Bio-demographic processes and genetic effects

Because the FHS is comprised of individuals from different generations (or, more broadly, from different birth cohorts), the FHS participants have different ages at the same time point. Genetic effects can be different at different chronological ages (e.g., [66] and references therein) and in different birth cohorts. Chronological age is a proxy of biological aging, whereas birth cohorts are a proxy of environmental exposures in which these cohorts grew up. Because aging ultimately affects lifespan, another important characteristic in aging cohorts is survival selection. The latter is, particularly, reflected in the distribution of ages at biospecimens collection [82]. Aging and environment represent two different mechanisms affecting linkage of genes with complex traits [7]. To gain some insights on these mechanisms, the associations should be evaluated in samples of appropriately matched individuals. To match the samples based on survival selection, individuals should be first matched by age at biospecimens collection. Then, individuals from different birth cohorts should be matched by age at a measurement of a factor of interest (TC in our case) as long as it varies during the individual’s life.

To meet those constraints, we focused on the FHSO and 3^rd^ Gen cohorts, because they included the largest number of individuals to match. Frequency distributions of age at biospecimens collection for each sex in these cohorts are shown in S2 Fig Because TC was measured discretely during follow-up (i.e., at examinations), we evaluated mean ages at each FHSO examination. The closest matches of mean age at TC measurement in the 3^rd^ Gen (50.1 years) were at the 6^th^ (48.6 years) and 7^th^ (51.4 years) FHSO examinations. We selected the 6^th^ examination because the estimates of genetic associations with TC at this examination resembled cumulative estimates over the life course shown in Fig 1.

## Ethics Statement

This study uses de-identified data from the FHS. The FHS data are available from the NHLBI through dbGaP. No new data were collected in this work. As such, this study does not require either ethics committee approval or written consent.

## Supporting Information

S1 Fig Significance of the associations of SNPs with TC across three FHS generations. Symbols show direction of the effect beta for the minor allele and -log_10_(p-value). Solid horizontal and dotted lines depict the level of nominal (p = 0.05) and suggestive effect (p = 0.1) significance. Numerical estimates are given in S2 Table.
(TIF)

S2 Fig Frequency distributions of age at biospecimens collection for men and women in the FHSO and 3^rd^ Gen cohorts. (TIF)

S1 Table Associations of 19 imputed SNPs with total cholesterol (TC) in different cohorts of the FHS participants. (PDF)

S2 Table Associations of 10 directly genotyped SNPs with total cholesterol (TC) in different cohorts of FHS participants. (PDF)

S3 Table Associations of SNPs with total cholesterol (TC) in more homogeneous samples of FHS participants. (PDF)

S4 Table Associations of total cholesterol (TC) with fasting in genotyped individuals in each Framingham cohort at different examinations. (PDF)

S5 Table Associations of total cholesterol (TC) with lipid-lowering treatment in genotyped individuals in each Framingham cohort at different examinations. (PDF)

S6 Table Associations of SNPs with total cholesterol (TC) measured at selected examinations in FHSO and 3^rd^ Gen cohorts of men and women combined. (PDF)

S7 Table Associations of SNPs with total cholesterol (TC) in younger and older sub-cohorts of each sex from FHSO and 3^rd^ Gen cohorts. (PDF)

S8 Table Antagonistic associations of rs2479409 with total cholesterol (TC). (PDF)
