# Supplementary material for: Birth Cohort, Age, and Sex Strongly Modulate Effects of Lipid Risk Alleles Identified in Genome-Wide Association Studies
Source: PLoS One. 2015 Aug 21;10(8):e0136319. doi: 10.1371/journal.pone.0136319 (PMC4546650; doi:10.1371/journal.pone.0136319)
Supplement: S1 Table — (PDF) [file pone.0136319.s003.pdf]

**S1 Table. Associations of 19 imputed SNPs with total cholesterol (TC) in different cohorts of the FHS participants**

| N | SNP | | Sex | Nat-ure* | All, N=8500 | | | FHS, N**=938 | | | FHSO, N**=3675 | | | 3rd Gen, N**=3887 | | |
|---|---|---|---|---|---|---|---|---|---|---|---|---|---|---|---|---|
| | Nature | FHS proxy | | | Beta | SE | p | Beta | SE | p | Beta | SE | p | Beta | SE | p |
| 1 | rs12027135 | rs926438 | M&W | - | -0.16 | 0.11 | 1.4E-01 | 0.02 | 0.24 | 9.4E-01 | -0.12 | 0.15 | 4.4E-01 | -0.19 | 0.18 | 3.0E-01 |
| | | | men | | -0.27 | 0.16 | 9.4E-02 | -0.08 | 0.39 | 8.4E-01 | -0.28 | 0.22 | 1.9E-01 | -0.21 | 0.27 | 4.4E-01 |
| | | | women | | 0.01 | 0.15 | 9.5E-01 | 0.12 | 0.31 | 7.0E-01 | 0.03 | 0.21 | 8.8E-01 | -0.22 | 0.24 | 3.6E-01 |
| 2 | rs2131925 | rs1748195 | M&W | - | -0.47 | 0.12 | 5.5E-05 | -0.71 | 0.26 | 5.7E-03 | -0.62 | 0.16 | 8.0E-05 | -0.26 | 0.19 | 1.8E-01 |
| | | | men | | -0.43 | 0.17 | 1.4E-02 | -0.61 | 0.41 | 1.4E-01 | -0.28 | 0.23 | 2.2E-01 | -0.48 | 0.29 | 1.0E-01 |
| | | | women | | -0.57 | 0.15 | 1.4E-04 | -0.79 | 0.32 | 1.4E-02 | -0.97 | 0.21 | 3.7E-06 | -0.11 | 0.25 | 6.7E-01 |
| 3 | rs7515577 | rs4970712 | M&W | - | -0.14 | 0.14 | 3.1E-01 | 0.03 | 0.30 | 9.3E-01 | -0.19 | 0.19 | 3.2E-01 | -0.23 | 0.22 | 3.1E-01 |
| | | | men | | -0.03 | 0.20 | 8.8E-01 | 0.08 | 0.46 | 8.7E-01 | -0.08 | 0.28 | 7.7E-01 | 0.04 | 0.34 | 9.2E-01 |
| | | | women | | -0.22 | 0.18 | 2.3E-01 | -0.05 | 0.39 | 9.0E-01 | -0.21 | 0.25 | 4.1E-01 | -0.49 | 0.29 | 9.6E-02 |
| 4 | rs629301 | rs599839 | M&W | - | -1.20 | 0.13 | 2.5E-20 | -0.92 | 0.29 | 1.4E-03 | -1.21 | 0.18 | 1.1E-11 | -1.17 | 0.21 | 5.6E-08 |
| | | | men | | -1.18 | 0.19 | 9.3E-10 | -0.86 | 0.43 | 4.4E-02 | -1.48 | 0.26 | 1.9E-08 | -0.88 | 0.33 | 7.4E-03 |
| | | | women | | -1.13 | 0.17 | 2.4E-11 | -0.90 | 0.37 | 1.6E-02 | -0.91 | 0.24 | 1.2E-04 | -1.40 | 0.28 | 4.9E-07 |
| 5 | rs514230 | rs553427 | M&W | - | 0.50 | 0.11 | 3.5E-06 | 0.65 | 0.24 | 7.4E-03 | 0.52 | 0.15 | 4.8E-04 | 0.43 | 0.18 | 1.7E-02 |
| | | | men | | 0.39 | 0.16 | 1.5E-02 | 0.36 | 0.38 | 3.5E-01 | 0.39 | 0.22 | 7.5E-02 | 0.60 | 0.27 | 2.8E-02 |
| | | | women | | 0.54 | 0.14 | 1.4E-04 | 0.75 | 0.31 | 1.4E-02 | 0.64 | 0.20 | 1.6E-03 | 0.32 | 0.23 | 1.7E-01 |
| 6 | rs1260326 | rs780094 | M&W | + | 0.61 | 0.11 | 2.3E-08 | 0.36 | 0.24 | 1.4E-01 | 0.70 | 0.15 | 3.2E-06 | 0.51 | 0.18 | 4.3E-03 |
| | | | men | | 0.47 | 0.16 | 3.6E-03 | 0.13 | 0.38 | 7.4E-01 | 0.79 | 0.22 | 3.0E-04 | 0.00 | 0.27 | 1.0E+00 |
| | | | women | | 0.67 | 0.14 | 2.6E-06 | 0.48 | 0.31 | 1.2E-01 | 0.54 | 0.20 | 6.7E-03 | 0.99 | 0.23 | 1.8E-05 |
| 7 | rs2290159 | rs7956 | M&W | - | -0.27 | 0.13 | 4.2E-02 | -0.37 | 0.28 | 2.0E-01 | -0.26 | 0.18 | 1.6E-01 | -0.24 | 0.22 | 2.7E-01 |
| | | | men | | -0.34 | 0.19 | 7.7E-02 | -0.37 | 0.45 | 4.1E-01 | -0.61 | 0.27 | 2.3E-02 | -0.04 | 0.32 | 9.1E-01 |
| | | | women | | -0.13 | 0.17 | 4.5E-01 | -0.31 | 0.36 | 4.0E-01 | 0.10 | 0.25 | 7.0E-01 | -0.39 | 0.28 | 1.7E-01 |
| 8 | rs12916 | rs3846663 | M&W | + | 0.52 | 0.11 | 3.9E-06 | 0.15 | 0.25 | 5.5E-01 | 0.53 | 0.15 | 6.4E-04 | 0.52 | 0.18 | 4.6E-03 |
| | | | men | | 0.32 | 0.17 | 5.4E-02 | 0.03 | 0.40 | 9.4E-01 | 0.26 | 0.23 | 2.6E-01 | 0.47 | 0.28 | 8.8E-02 |
| | | | women | | 0.62 | 0.15 | 2.2E-05 | 0.25 | 0.31 | 4.2E-01 | 0.69 | 0.20 | 7.2E-04 | 0.50 | 0.24 | 3.4E-02 |
| 9 | rs6882076 | rs1501908 | M&W | - | -0.40 | 0.11 | 3.7E-04 | -0.60 | 0.25 | 1.7E-02 | -0.19 | 0.15 | 2.1E-01 | -0.66 | 0.18 | 3.8E-04 |
| | | | men | | -0.23 | 0.17 | 1.7E-01 | -0.69 | 0.40 | 8.3E-02 | -0.14 | 0.22 | 5.3E-01 | -0.41 | 0.28 | 1.4E-01 |
| | | | women | | -0.55 | 0.15 | 1.7E-04 | -0.65 | 0.31 | 4.0E-02 | -0.23 | 0.21 | 2.8E-01 | -0.79 | 0.24 | 1.0E-03 |
| 10 | rs3757354 | rs7759222 | M&W | - | -0.39 | 0.13 | 4.2E-03 | -0.79 | 0.31 | 1.0E-02 | -0.17 | 0.19 | 3.6E-01 | -0.39 | 0.22 | 7.5E-02 |
| | | | men | | -0.33 | 0.20 | 9.5E-02 | -0.86 | 0.47 | 6.6E-02 | 0.03 | 0.27 | 9.3E-01 | -0.62 | 0.33 | 6.1E-02 |
| | | | women | | -0.38 | 0.18 | 3.3E-02 | -0.70 | 0.40 | 7.5E-02 | -0.35 | 0.25 | 1.7E-01 | -0.20 | 0.29 | 4.9E-01 |
| 11 | rs9987289 | rs4841133 | M&W | - | -0.92 | 0.20 | 6.7E-06 | -0.87 | 0.45 | 5.5E-02 | -0.78 | 0.28 | 6.1E-03 | -1.25 | 0.33 | 1.7E-04 |
| | | | men | | -0.82 | 0.31 | 7.3E-03 | -0.92 | 0.77 | 2.3E-01 | -0.24 | 0.41 | 5.6E-01 | -1.80 | 0.51 | 4.2E-04 |
| | | | women | | -0.89 | 0.27 | 8.3E-04 | -0.78 | 0.55 | 1.5E-01 | -1.07 | 0.39 | 5.8E-03 | -0.74 | 0.43 | 8.4E-02 |
| 12 | rs1495741 | rs1495743 | M&W | + | 0.26 | 0.13 | 4.7E-02 | 0.36 | 0.27 | 1.9E-01 | 0.24 | 0.18 | 1.8E-01 | 0.38 | 0.22 | 8.7E-02 |

| | | | | | | | | | | | | | | | | |
|---|---|---|---|---|---|---|---|---|---|---|---|---|---|---|---|---|
| | | | men | | 0.50 | 0.19 | 8.4E-03 | 0.71 | 0.41 | 8.6E-02 | 0.33 | 0.26 | 2.0E-01 | 0.45 | 0.34 | 1.9E-01 |
| | | | women | | 0.21 | 0.17 | 2.2E-01 | 0.18 | 0.36 | 6.1E-01 | 0.26 | 0.24 | 2.9E-01 | 0.42 | 0.29 | 1.5E-01 |
| 13 | rs2081687 | rs6985620 | M&W | + | 0.33 | 0.11 | 3.6E-03 | 0.47 | 0.26 | 6.7E-02 | 0.38 | 0.16 | 1.5E-02 | -0.03 | 0.19 | 8.7E-01 |
| | | | men | | 0.37 | 0.17 | 2.8E-02 | 0.58 | 0.40 | 1.4E-01 | 0.50 | 0.23 | 2.7E-02 | 0.00 | 0.29 | 1.0E+00 |
| | | | women | | 0.28 | 0.15 | 5.9E-02 | 0.40 | 0.33 | 2.3E-01 | 0.26 | 0.21 | 2.1E-01 | -0.09 | 0.24 | 7.2E-01 |
| 14 | rs2954029 | rs2980875 | M&W | - | -0.49 | 0.11 | 1.3E-05 | -0.33 | 0.26 | 2.1E-01 | -0.47 | 0.15 | 1.9E-03 | -0.54 | 0.19 | 4.5E-03 |
| | | | men | | -0.52 | 0.17 | 2.0E-03 | -0.06 | 0.42 | 8.8E-01 | -0.49 | 0.22 | 2.9E-02 | -0.75 | 0.29 | 8.5E-03 |
| | | | women | | -0.46 | 0.15 | 1.7E-03 | -0.52 | 0.33 | 1.1E-01 | -0.44 | 0.20 | 2.7E-02 | -0.36 | 0.25 | 1.4E-01 |
| 15 | rs2255141 | rs2792751 | M&W | + | 0.19 | 0.12 | 1.0E-01 | 0.43 | 0.27 | 1.1E-01 | 0.09 | 0.16 | 5.8E-01 | 0.32 | 0.19 | 9.4E-02 |
| | | | men | | 0.31 | 0.18 | 7.6E-02 | 0.25 | 0.41 | 5.5E-01 | 0.24 | 0.24 | 3.3E-01 | 0.42 | 0.29 | 1.6E-01 |
| | | | women | | 0.12 | 0.15 | 4.4E-01 | 0.51 | 0.35 | 1.4E-01 | 0.05 | 0.21 | 8.0E-01 | 0.18 | 0.25 | 4.6E-01 |
| 16 | rs174546 | rs174547 | M&W | - | -0.42 | 0.12 | 3.4E-04 | 0.03 | 0.26 | 9.2E-01 | -0.61 | 0.16 | 1.7E-04 | -0.22 | 0.19 | 2.6E-01 |
| | | | men | | -0.35 | 0.17 | 4.1E-02 | 0.09 | 0.41 | 8.3E-01 | -0.62 | 0.23 | 7.9E-03 | 0.12 | 0.29 | 6.9E-01 |
| | | | women | | -0.50 | 0.15 | 1.1E-03 | 0.09 | 0.33 | 7.9E-01 | -0.57 | 0.22 | 8.5E-03 | -0.48 | 0.25 | 5.6E-02 |
| 17 | rs7241918 | rs7240405 | M&W | - | -0.28 | 0.15 | 6.1E-02 | -0.18 | 0.34 | 6.0E-01 | -0.22 | 0.20 | 2.7E-01 | -0.17 | 0.24 | 4.9E-01 |
| | | | men | | -0.40 | 0.22 | 7.4E-02 | -0.26 | 0.54 | 6.3E-01 | -0.54 | 0.30 | 7.1E-02 | -0.01 | 0.37 | 9.9E-01 |
| | | | women | | -0.12 | 0.19 | 5.3E-01 | -0.08 | 0.42 | 8.6E-01 | 0.09 | 0.27 | 7.4E-01 | -0.28 | 0.31 | 3.7E-01 |
| 18 | rs2277862 | rs6119625 | M&W | - | -0.35 | 0.15 | 1.8E-02 | 0.30 | 0.33 | 3.7E-01 | -0.37 | 0.20 | 6.8E-02 | -0.53 | 0.24 | 3.0E-02 |
| | | | men | | -0.36 | 0.22 | 9.9E-02 | 0.37 | 0.51 | 4.7E-01 | -0.56 | 0.29 | 5.7E-02 | -0.36 | 0.37 | 3.4E-01 |
| | | | women | | -0.32 | 0.19 | 1.0E-01 | 0.26 | 0.43 | 5.5E-01 | -0.14 | 0.27 | 6.1E-01 | -0.68 | 0.32 | 3.2E-02 |
| 19 | rs2902940 | rs2143877 | M&W | - | -0.13 | 0.12 | 2.7E-01 | -0.16 | 0.26 | 5.5E-01 | -0.32 | 0.17 | 5.1E-02 | 0.17 | 0.20 | 3.9E-01 |
| | | | men | | -0.18 | 0.18 | 3.1E-01 | -0.06 | 0.41 | 8.8E-01 | -0.71 | 0.24 | 3.1E-03 | 0.51 | 0.30 | 8.5E-02 |
| | | | women | | -0.09 | 0.16 | 5.8E-01 | -0.24 | 0.33 | 4.7E-01 | 0.03 | 0.22 | 9.0E-01 | -0.18 | 0.26 | 4.8E-01 |

Proxy SNPs were selected as those which were present on the FHS Affymetrix array and were in strong linkage disequilibrium (LD; $r^2$>0.9) to those reported in the Nature meta-analysis. LD was available from the 1000 Genomes project for CEU population. Nineteen SNPs which were not in LD between each other and passed quality control (Hardy Weinberg Equilibrium p>0.01, Mendel's errors <2%, and call rate >90%) were retained. These SNPs were used to verify whether discordance of the effects observed for the directly genotyped 10 SNPs was also characteristic for the proxy SNPs.

*Column "Nature" shows direction of the effect from the Nature meta-analysis. The effect size is not shown because it is not directly comparable with the effect size evaluated for log transformed TC (i.e., $100\times\log_{10}$(TC)) in the FHS.

Sign of the effect indicates direction in additive genetic model with minor allele considered as an effect allele, e.g., plus sign implies increasing TC values for minor allele carriers. M&W denotes men and women.

**N denotes maximal number of individuals across SNPs used in the analyses in each cohort at baseline.

FHS is Framingham Heart Study (FHS) original cohort; FHSO is FHS Offspring cohort; 3rd Gen is FHS 3rd generation cohort. "All" denotes pooled sample of all FHS participants.

The associations of SNPs with TC in the 3rd Gen cohort were evaluated for TC measured at baseline. In all other samples we evaluated cumulative associations of SNPs with TC over the selected examinations (16 in the FHS and seven in the FHSO).

Table S1 shows that the effects are markedly different (and are of even antagonistic signs, e.g., rs6119625, rs2143877) in different demographic cohorts defined by the FHS generations and sex for all, except two (rs599839 and rs553427), proxy SNPs.
