# Supplementary material for: Birth Cohort, Age, and Sex Strongly Modulate Effects of Lipid Risk Alleles Identified in Genome-Wide Association Studies
Source: PLoS One. 2015 Aug 21;10(8):e0136319. doi: 10.1371/journal.pone.0136319 (PMC4546650; doi:10.1371/journal.pone.0136319)
Supplement: S2 Table — (PDF) [file pone.0136319.s004.pdf]

S2 Table. Associations of 10 directly genotyped SNPs with total cholesterol (TC) in different cohorts of FHS participants

N	SNP	Sex	All cohorts, N=8500			FHS, 16 examinations, N*=938			FHSO, 7 examinations N*=3675			3rd Gen N*=3887		
			Beta**	SE	p	Beta**	SE	p	Beta**	SE	p	Beta**	SE	p
1	rs2479409	M&W	0.10	0.11	3.5E-01	0.27	0.24	2.5E-01	0.25	0.16	1.1E-01	-0.25	0.18	1.7E-01
		men	0.01	0.17	9.4E-01	0.27	0.36	4.5E-01	0.31	0.23	1.8E-01	-0.53	0.28	5.9E-02
		women	0.21	0.15	1.6E-01	0.40	0.31	2.0E-01	0.19	0.21	3.8E-01	-0.03	0.24	8.9E-01
2	rs3177928	M&W	0.44	0.16	5.7E-03	0.32	0.35	3.5E-01	0.40	0.23	8.1E-02	0.82	0.26	1.6E-03
		men	0.71	0.24	3.2E-03	-0.17	0.56	7.7E-01	0.96	0.33	4.1E-03	0.95	0.40	1.8E-02
		women	0.24	0.21	2.5E-01	0.61	0.43	1.6E-01	-0.05	0.30	8.7E-01	0.78	0.33	1.9E-02
3	rs1800562	M&W	-0.51	0.23	2.7E-02	-0.03	0.52	9.5E-01	-0.18	0.32	5.6E-01	-1.39	0.37	2.2E-04
		men	-0.69	0.34	4.3E-02	-0.59	0.83	4.8E-01	-0.17	0.46	7.1E-01	-1.52	0.56	6.9E-03
		women	-0.44	0.30	1.5E-01	0.38	0.65	5.6E-01	-0.19	0.42	6.6E-01	-1.16	0.49	1.8E-02
4	rs9488822	M&W	-0.21	0.11	5.8E-02	-0.60	0.23	1.0E-02	-0.15	0.15	3.5E-01	-0.13	0.18	4.7E-01
		men	-0.10	0.16	5.4E-01	-0.36	0.37	3.4E-01	-0.25	0.22	2.5E-01	0.33	0.28	2.4E-01
		women	-0.32	0.15	2.6E-02	-0.63	0.29	3.2E-02	-0.07	0.21	7.5E-01	-0.49	0.24	4.4E-02
5	rs1564348	M&W	0.57	0.15	9.9E-05	0.04	0.30	8.8E-01	0.75	0.21	3.0E-04	0.38	0.25	1.3E-01
		men	0.39	0.22	7.1E-02	0.00	0.49	1.0E+00	0.58	0.29	4.6E-02	0.08	0.37	8.3E-01
		women	0.60	0.19	2.0E-03	0.06	0.38	8.7E-01	0.75	0.29	9.6E-03	0.66	0.33	4.3E-02
6	rs10128711	M&W	-0.34	0.13	7.2E-03	-0.27	0.26	3.0E-01	-0.37	0.17	3.3E-02	-0.42	0.21	4.7E-02
		men	-0.25	0.19	1.8E-01	-0.26	0.41	5.2E-01	-0.35	0.26	1.7E-01	-0.25	0.32	4.5E-01
		women	-0.43	0.16	9.3E-03	-0.24	0.34	4.7E-01	-0.37	0.23	1.1E-01	-0.54	0.27	4.8E-02
7	rs11220462	M&W	0.31	0.16	4.9E-02	0.69	0.37	6.1E-02	0.14	0.22	5.1E-01	0.49	0.26	5.4E-02
		men	0.17	0.23	4.5E-01	1.06	0.58	6.9E-02	-0.06	0.30	8.4E-01	0.59	0.39	1.3E-01
		women	0.49	0.21	1.8E-02	0.55	0.46	2.3E-01	0.34	0.30	2.6E-01	0.57	0.33	8.5E-02
8	rs3764261	M&W	0.32	0.12	7.1E-03	0.07	0.26	8.0E-01	0.12	0.17	5.0E-01	0.59	0.19	2.1E-03
		men	0.51	0.18	3.8E-03	0.31	0.40	4.4E-01	0.14	0.25	5.7E-01	0.84	0.29	3.5E-03
		women	0.08	0.16	6.1E-01	-0.23	0.33	4.9E-01	0.09	0.23	7.0E-01	0.25	0.25	3.2E-01
9	rs7206971	M&W	0.09	0.11	3.8E-01	0.05	0.22	8.1E-01	0.08	0.15	6.0E-01	0.31	0.18	9.0E-02
		men	0.06	0.16	7.2E-01	0.28	0.36	4.3E-01	0.10	0.22	6.4E-01	-0.02	0.27	9.4E-01
		women	0.15	0.14	2.9E-01	-0.15	0.28	5.9E-01	0.11	0.20	5.9E-01	0.67	0.23	4.3E-03
10	rs1800961	M&W	-1.66	0.31	5.8E-08	-1.69	0.63	7.4E-03	-1.55	0.42	2.6E-04	-1.35	0.52	8.7E-03
		men	-1.37	0.44	1.7E-03	-0.73	0.97	4.5E-01	-1.01	0.59	8.9E-02	-1.96	0.75	9.0E-03
		women	-2.00	0.41	1.4E-06	-2.40	0.82	3.5E-03	-2.17	0.59	2.6E-04	-0.64	0.69	3.6E-01

[*]N denotes maximal number of individuals across SNPs used in the analyses in each cohort at baseline.
[**]The effect size beta is evaluated for $100 \times \log_{10}$(TC)
Sign of beta indicates direction of the effect in additive genetic model with minor allele considered as an effect allele, e.g., plus sign implies increasing TC values for minor allele carriers
M&W denotes men and women
FHS is Framingham Heart Study (FHS) original cohort; FHSO is FHS Offspring cohort; 3rd Gen is FHS 3rd generation cohort
"All cohorts" denotes pooled sample of all FHS participants
The associations of SNPs with TC in the 3rd Gen cohort were evaluated for TC measured at baseline. In all other samples we evaluated cumulative associations of SNPs with total cholesterol (TC) over the selected examinations (16 in the FHS and seven in the FHSO)
