# Supplementary material for: Birth Cohort, Age, and Sex Strongly Modulate Effects of Lipid Risk Alleles Identified in Genome-Wide Association Studies
Source: PLoS One. 2015 Aug 21;10(8):e0136319. doi: 10.1371/journal.pone.0136319 (PMC4546650; doi:10.1371/journal.pone.0136319)
Supplement: S3 Table — (PDF) [file pone.0136319.s005.pdf]

S3 Table. Associations of SNPs with total cholesterol (TC) in more homogeneous samples of FHS participants

SNP ID	S-subsample						W-subsample						$I^2_{S\text{-}W}$, %	All
	Cohorts	N*	Beta**	SE	p	$N_{5E\text{-}8}$	Cohorts	N*	Beta**	SE	p	$N_{5E\text{-}8}$		$N_{5E\text{-}8}$
Selected														
rs2479409	C1aC2aC3w	6,645	0.20	0.12	8.9E-02	154,891	C3m	1,819	-0.53	0.28	5.9E-02	22,039	82.6	619,623
rs3177928	C1wC2mC3a	6,151	0.74	0.19	9.9E-05	21,070	C1mC2w	2,276	-0.10	0.28	7.2E-01	$>10^6$	83.8	59,633
rs1800562	C1mC3a	4,279	-1.45	0.37	8.2E-05	11,706	C1wC2a	4,222	-0.07	0.29	8.2E-01	$>10^6$	88.4	94,766
rs9488822	C1aC3w	2,962	-0.56	0.17	1.3E-03	19,002	C2aC3m	5,425	-0.05	0.14	7.1E-01	$>10^6$	81.4	135,245
rs1564348	C2aC3w	5,720	0.74	0.18	3.1E-05	18,873	C1aC3m	2,757	0.16	0.24	5.1E-01	404,114	73.3	31,823
rs11220462	C1aC2wC3a	6,709	0.48	0.18	8.2E-03	53,341	C2m	1,751	-0.06	0.30	8.4E-01	$>10^6$	58.0	127,912
rs3764261	C3m	1,815	0.84	0.29	3.5E-03	9,364	C1aC2aC3w	6,469	0.17	0.13	1.9E-01	229,092	77.5	64,642
rs7206971	C3w	2,061	0.67	0.24	4.3E-03	12,375	C1aC2aC3m	6,364	0.02	0.12	8.7E-01	$>10^6$	83.0	686,911
rs1800961	C1wC2wC3m	4,271	-2.23	0.42	1.1E-07	9,589	C1mC2mC3w	4,213	-1.03	0.43	1.7E-02	45,020	74.9	17,320
Not selected														
SNP ID	Sample	N*	Beta**	SE	p	I^2_S, %	Sample	N*	Beta**	SE	p	I^2_W, %	$I^2_{S\text{-}W}$, %	
rs2479409	C1aC2a	4,577	0.24	0.14	7.7E-02	0.0	C3a	3,887	-0.25	0.18	1.7E-01	46.0	78.3	
rs1800562	C3a	3,888	-1.39	0.38	2.2E-04	0.0	C1aC2a	4,613	-0.07	0.28	7.9E-01	0.0	87.2	
rs9488822	C1wC3w	2,584	-0.58	0.19	2.7E-03	0.0	C1mC2aC3m	5,803	-0.07	0.13	6.0E-01	0.0	73.2	
rs7206971	C1mC3w	2,433	0.38	0.22	7.5E-02	0.0	C1wC2aC3m	5,992	0.02	0.12	8.8E-01	0.0	51.5	

*The number of individuals used in the analyses in each sample at baseline examinations

**The effect size beta is evaluated for $100\times\log_{10}$(TC); SE denotes standard error

Sign of beta indicates direction of the effect in additive genetic model with minor allele considered as an effect allele, e.g., plus sign implies increasing TC values for minor allele carriers

The S-subsample includes the FHS demographic cohorts (see notations below), which provide the strongest support to the results of the Nature meta-analysis and have homogeneous effects, i.e., $I^2=0$ for the effects between cohorts included into this subsample. The W-subsample was selected in the same manner as the S-subsample (i.e., requiring $I^2=0$ for the effects between demographic cohorts included into the W-subsample) but it provides the weakest support to the results in the Nature meta-analysis. The S- and W-subsamples were selected based on the results presented in Figure 1 for all SNPs except rs10128711 (for which the effects were homogeneous across all demographic cohorts) to maximize heterogeneity in the effects between these S- and W-subsamples (see column $I^2_{S\text{-}W}$). For unclear situations, we provide the results for alternative stratifications (see bottom of the Table, "***Not selected***"). These results show that heterogeneity of the effects between the S- and W-subsamples is smaller for alternative scenarios and, thus, they were not selected.

Demographic cohorts refer to men and/or women from different generations as denoted in columns "Cohorts", i.e., letter "C" denotes cohort with the number indicating: 1=original cohort, 2=Offspring cohort, and 3=3rd Generation cohort. Letters "a", "m", and "w" denote pooled sample of men and women, sample of men, and sample of women, respectively.

$N_{5E\text{-}8}$ denotes the sample size which is needed to achieve genome-wide significance (i.e., $p=5.0\times10^{-8}$). Column "All" shows the sample size estimates based on the effect sizes in the pooled sample of all FHS participants provided in S2 Table.
