# Supplementary material for: Birth Cohort, Age, and Sex Strongly Modulate Effects of Lipid Risk Alleles Identified in Genome-Wide Association Studies
Source: PLoS One. 2015 Aug 21;10(8):e0136319. doi: 10.1371/journal.pone.0136319 (PMC4546650; doi:10.1371/journal.pone.0136319)
Supplement: S4 Table — (PDF) [file pone.0136319.s006.pdf]

S4 Table. Associations of total cholesterol (TC) with fasting in genotyped individuals in each Framingham cohort at different examinations

Cohort	Exam	N_{total}	N_{fast}	% fasting	Beta*	SE	P-value
FHS	7	1376	183	12.9	0.03	0.60	9.6E-01
	8	1412	98	6.9	2.20	0.77	4.4E-03
	9	1403	61	4.3	3.85	0.99	1.2E-04
	10	1316	265	20.1	-0.21	0.52	6.9E-01
FHSO	1	3677	3661	99.1	-1.78	1.92	3.5E-01
	2	3033	3020	98.7	-2.58	2.07	2.1E-01
	3	3073	2913	93.2	0.29	0.64	6.5E-01
	4	3273	3032	90.4	-0.20	0.53	7.1E-01
	5	3316	3210	96.6	0.95	0.76	2.1E-01
	6	3208	3076	95.7	0.78	0.71	2.7E-01
	7	3131	2911	92.5	1.49	0.55	6.5E-03
3rd Gen	1	3886	3747	96.4	1.58	0.71	2.7E-02

*The effect size beta is evaluated for $100 \times \log_{10}(TC)$

N_{total} denotes the total number of individuals used in the analyses; N_{fast} denotes the number of fasting individuals among them

Sign for beta indicates direction of the effect, e.g., plus sign implies increasing TC values for individuals who have been fasting (12+ hours) compared to the others

SE denotes standard error

FHS is Framingham Heart Study (FHS) original cohort; FHSO is FHS Offspring cohort; 3rd Gen is FHS 3rd generation cohort
