# Supplementary material for: Birth Cohort, Age, and Sex Strongly Modulate Effects of Lipid Risk Alleles Identified in Genome-Wide Association Studies
Source: PLoS One. 2015 Aug 21;10(8):e0136319. doi: 10.1371/journal.pone.0136319 (PMC4546650; doi:10.1371/journal.pone.0136319)
Supplement: S5 Table — (PDF) [file pone.0136319.s007.pdf]

S5 Table. Associations of total cholesterol (TC) with lipid-lowering treatment in genotyped individuals in each Framingham cohort at different examinations

Cohort	Exam	N_{total}	N_{med}	% treated	Beta*	SE	P-value
FHS	10	1311	15	1.1	3.68	1.97	6.2E-02
	13	1433	34	2.4	0.12	1.31	9.3E-01
	14	1430	40	2.8	2.47	1.17	3.6E-02
	15	1412	44	3.1	2.46	1.12	2.8E-02
	20	892	45	5.0	0.74	1.24	5.5E-01
	22	725	64	8.8	1.30	1.01	2.0E-01
	24	542	72	13.3	-0.14	0.99	8.9E-01
	26	318	99	31.0	-3.15	1.04	2.9E-03
FHSO	1	3679	20	0.5	4.60	1.71	7.3E-03
	2	3057	33	1.1	5.81	1.31	9.4E-06
	3	3125	30	1.0	5.48	1.42	1.2E-04
	4	3345	130	3.9	4.20	0.71	3.5E-09
	5	3324	254	7.6	1.67	0.51	1.0E-03
	6	3213	445	13.8	-1.31	0.42	1.7E-03
	7	3147	664	21.1	-4.31	0.34	4.8E-35
3rd Gen	1	3886	328	8.4	-2.09	0.46	5.5E-06

*The effect size beta is evaluated for $100 \times \log_{10}(TC)$

N_{total} denotes the total number of individuals used in the analyses; N_{med} denotes the number of individuals, who have been on lipid-lowering treatment, among them

Sign for beta indicates direction of the effect, e.g., plus sign implies increasing TC values for individuals who have been on lipid-lowering treatment compared to the others

SE denotes standard error

FHS is Framingham Heart Study (FHS) original cohort; FHSO is FHS Offspring cohort; 3rd Gen is FHS 3rd generation cohort
