# Supplementary material for: Birth Cohort, Age, and Sex Strongly Modulate Effects of Lipid Risk Alleles Identified in Genome-Wide Association Studies
Source: PLoS One. 2015 Aug 21;10(8):e0136319. doi: 10.1371/journal.pone.0136319 (PMC4546650; doi:10.1371/journal.pone.0136319)
Supplement: S6 Table — (PDF) [file pone.0136319.s008.pdf]

S6 Table. Associations of SNPs with total cholesterol (TC) measured at selected examinations in FHSO and 3rd Gen cohorts of men and women combined

SNP	Cohort	Exam	Sample	N	Beta*	SE	P-value
rs2479409	FHSO	6	all	3210	0.33	0.21	1.2E-01
		7	all	3144	0.17	0.21	4.3E-01
	3rd Gen	1	all	3887	-0.25	0.18	1.7E-01
	FHSO	6	no medications	2764	0.33	0.23	1.4E-01
		7	no medications	2479	0.17	0.22	4.4E-01
	3rd Gen	1	no medications	3557	-0.22	0.19	2.3E-01
rs3177928	FHSO	6	all	3181	0.61	0.31	4.7E-02
		7	all	3116	0.60	0.30	4.8E-02
	3rd Gen	1	all	3866	0.82	0.26	1.6E-03
	FHSO	6	no medications	2738	0.65	0.33	4.5E-02
		7	no medications	2455	0.87	0.33	7.5E-03
	3rd Gen	1	no medications	3538	0.85	0.26	1.3E-03
rs1800562	FHSO	6	all	3207	-0.31	0.43	4.8E-01
		7	all	3140	-0.78	0.43	6.9E-02
	3rd Gen	1	all	3888	-1.39	0.37	2.2E-04
	FHSO	6	no medications	2761	-0.58	0.45	2.0E-01
		7	no medications	2477	-0.82	0.47	7.9E-02
	3rd Gen	1	no medications	3558	-1.12	0.38	3.1E-03
rs9488822	FHSO	6	all	3158	-0.20	0.21	3.5E-01
		7	all	3089	-0.04	0.21	8.3E-01
	3rd Gen	1	all	3878	-0.13	0.18	4.7E-01
	FHSO	6	no medications	2723	-0.15	0.22	5.1E-01
		7	no medications	2435	0.09	0.22	6.7E-01
	3rd Gen	1	no medications	3548	-0.26	0.19	1.6E-01
rs1564348	FHSO	6	all	3187	0.84	0.28	2.6E-03
		7	all	3122	0.49	0.28	7.9E-02
	3rd Gen	1	all	3886	0.38	0.25	1.3E-01
	FHSO	6	no medications	2746	0.94	0.30	1.6E-03
		7	no medications	2462	0.67	0.30	2.7E-02
	3rd Gen	1	no medications	3556	0.41	0.25	1.0E-01
rs10128711	FHSO	6	all	3196	-0.37	0.24	1.2E-01
		7	all	3134	-0.12	0.23	6.1E-01
	3rd Gen	1	all	3864	-0.42	0.21	4.7E-02
	FHSO	6	no medications	2752	-0.48	0.25	5.8E-02
		7	no medications	2473	-0.39	0.25	1.1E-01
	3rd Gen	1	no medications	3537	-0.26	0.22	2.3E-01
rs11220462	FHSO	6	all	3198	-0.10	0.29	7.4E-01
		7	all	3133	-0.24	0.29	4.0E-01
	3rd Gen	1	all	3883	0.49	0.26	5.4E-02
	FHSO	6	no medications	2755	-0.17	0.31	5.8E-01
		7	no medications	2472	-0.27	0.31	3.8E-01
	3rd Gen	1	no medications	3553	0.47	0.26	7.2E-02

rs3764261	FHSO	6	all	3095	0.19	0.23	4.2E-01
		7	all	3025	0.49	0.23	3.4E-02
	3rd Gen	1	all	3878	0.59	0.19	2.1E-03
	FHSO	6	no medications	2660	0.06	0.25	8.2E-01
		7	no medications	2385	0.40	0.25	1.1E-01
	3rd Gen	1	no medications	3548	0.56	0.19	4.1E-03
rs7206971	FHSO	6	all	3191	0.36	0.20	7.8E-02
		7	all	3125	0.15	0.20	4.5E-01
	3rd Gen	1	all	3870	0.31	0.18	9.0E-02
	FHSO	6	no medications	2747	0.22	0.22	3.0E-01
		7	no medications	2464	0.21	0.22	3.3E-01
	3rd Gen	1	no medications	3542	0.31	0.18	8.8E-02
rs1800961	FHSO	6	all	3209	-1.71	0.57	2.6E-03
		7	all	3142	-1.86	0.57	1.1E-03
	3rd Gen	1	all	3885	-1.35	0.52	8.7E-03
	FHSO	6	no medications	2763	-2.01	0.60	8.3E-04
		7	no medications	2477	-1.83	0.61	2.8E-03
	3rd Gen	1	no medications	3556	-1.12	0.52	3.1E-02

*The effect size beta is evaluated for $100\times\log_{10}(TC)$

Sign of beta indicates direction of the effect in additive genetic model with minor allele considered as an effect allele, e.g., plus sign implies increasing TC values for minor allele carriers

FHSO is Framingham Heart Study (FHS) Offspring cohort; 3rd Gen is FHS 3rd generation cohort

This table shows the associations of each SNP with TC measured at 6th and 7th FHSO and baseline 3rd Gen examinations at which lipid-lowering therapy was associated with lower TC levels (see S5 Table)

Column “Sample”: “all” indicates the entire sample at a given examination; “no medications” indicates the estimates for samples with individuals who are known to be on lipid-lowering therapy excluded
