# Supplementary material for: Birth Cohort, Age, and Sex Strongly Modulate Effects of Lipid Risk Alleles Identified in Genome-Wide Association Studies
Source: PLoS One. 2015 Aug 21;10(8):e0136319. doi: 10.1371/journal.pone.0136319 (PMC4546650; doi:10.1371/journal.pone.0136319)
Supplement: S7 Table — (PDF) [file pone.0136319.s009.pdf]

S7 Table. Associations of SNPs with total cholesterol (TC) in younger and older sub-cohorts of each sex from FHSO and 3rd Gen cohorts

SNP	Sex	3rd Gen					FHSO				
		Age	N	Beta*	SE	p	Age	N	Beta*	SE	p
rs2479409	M	≤40	852	-0.89	0.41	3.1E-02	40-60	740	0.48	0.44	2.8E-01
	M	40-60	967	-0.33	0.37	3.8E-01	>60	767	0.70	0.45	1.2E-01
	W	≤40	997	-0.89	0.33	8.0E-03	40-60	873	0.17	0.40	6.7E-01
	W	40-60	1071	0.81	0.33	1.6E-02	>60	830	0.18	0.38	6.3E-01
rs3177928	M	≤40	849	1.41	0.58	1.5E-02	40-60	735	0.85	0.65	2.0E-01
	M	40-60	964	0.58	0.53	2.8E-01	>60	761	1.70	0.65	9.1E-03
	W	≤40	990	1.09	0.46	1.7E-02	40-60	865	0.50	0.55	3.6E-01
	W	40-60	1063	0.41	0.48	3.9E-01	>60	820	-0.44	0.57	4.4E-01
rs1800562	M	≤40	852	-1.85	0.80	2.2E-02	40-60	739	0.07	0.94	9.4E-01
	M	40-60	967	-1.53	0.75	4.1E-02	>60	767	-0.35	0.90	7.0E-01
	W	≤40	998	-1.61	0.67	1.6E-02	40-60	872	-0.87	0.79	2.7E-01
	W	40-60	1071	-0.84	0.73	2.5E-01	>60	829	0.24	0.78	7.6E-01
rs9488822	M	≤40	850	0.30	0.41	4.6E-01	40-60	727	0.44	0.44	3.1E-01
	M	40-60	967	0.40	0.36	2.8E-01	>60	758	-0.53	0.42	2.1E-01
	W	≤40	996	-0.12	0.36	7.4E-01	40-60	862	-0.51	0.40	2.0E-01
	W	40-60	1065	-0.72	0.33	2.9E-02	>60	811	-0.15	0.39	7.1E-01
rs1564348	M	≤40	851	0.04	0.56	9.4E-01	40-60	732	0.22	0.57	6.9E-01
	M	40-60	967	-0.03	0.48	9.5E-01	>60	762	1.22	0.57	3.2E-02
	W	≤40	997	0.13	0.45	7.7E-01	40-60	866	1.02	0.54	6.1E-02
	W	40-60	1071	1.17	0.47	1.3E-02	>60	827	0.62	0.52	2.4E-01
rs10128711	M	≤40	846	-0.12	0.48	8.1E-01	40-60	734	-0.24	0.52	6.5E-01
	M	40-60	962	-0.25	0.42	5.5E-01	>60	765	-0.46	0.50	3.5E-01
	W	≤40	992	-1.14	0.40	4.2E-03	40-60	869	0.11	0.44	8.0E-01
	W	40-60	1064	0.04	0.38	9.3E-01	>60	828	-0.64	0.43	1.3E-01
rs11220462	M	≤40	850	0.48	0.58	4.1E-01	40-60	738	-0.79	0.59	1.8E-01
	M	40-60	966	0.41	0.50	4.2E-01	>60	764	0.20	0.60	7.4E-01
	W	≤40	997	0.61	0.48	2.0E-01	40-60	869	-0.44	0.56	4.4E-01
	W	40-60	1070	0.56	0.47	2.3E-01	>60	827	0.59	0.53	2.7E-01
rs3764261	M	≤40	851	0.95	0.41	2.1E-02	40-60	714	0.40	0.49	4.1E-01
	M	40-60	964	1.00	0.38	9.5E-03	>60	741	-0.14	0.48	7.8E-01
	W	≤40	997	0.16	0.35	6.4E-01	40-60	836	0.12	0.46	8.0E-01
	W	40-60	1066	0.33	0.35	3.6E-01	>60	804	0.17	0.42	6.9E-01
rs7206971	M	≤40	847	0.49	0.40	2.2E-01	40-60	735	-0.08	0.42	8.5E-01
	M	40-60	962	-0.56	0.36	1.2E-01	>60	765	0.60	0.42	1.6E-01
	W	≤40	993	0.78	0.33	1.9E-02	40-60	867	0.54	0.39	1.7E-01
	W	40-60	1068	0.56	0.33	9.2E-02	>60	824	0.24	0.37	5.1E-01
rs1800961	M	≤40	852	-0.54	1.04	6.0E-01	40-60	739	-3.16	1.09	4.0E-03
	M	40-60	965	-2.79	1.04	7.6E-03	>60	767	-0.09	1.22	9.4E-01
	W	≤40	997	-1.03	0.91	2.6E-01	40-60	873	-2.80	1.04	7.5E-03
	W	40-60	1071	0.08	1.06	9.4E-01	>60	830	-0.25	1.15	8.3E-01

*The effect size beta is evaluated for $100\times\log_{10}(TC)$
Sign of beta indicates direction of the effect in additive genetic model with minor allele considered as an effect allele, e.g., plus sign implies increasing TC values for minor allele carriers
Letter “M” denotes men and letter “W) denotes women; N denotes sample size
Genetic association in the FHSO cohorts were evaluated at the 6th examination (see Methods, “**Bio-demographic processes and genetic effects**”)
Column “Age” shows stratification into younger and older sub-cohorts as defined by median cut-off for age at biospecimens collection, i.e., 40 years in the 3rd Gen and 60 years in the FHSO. There were virtually no individuals aged older than 60 years in the 3rd Gen and younger than 40 years in the FHSO (see S1 Fig.).
