# Supplementary material for: Birth Cohort, Age, and Sex Strongly Modulate Effects of Lipid Risk Alleles Identified in Genome-Wide Association Studies
Source: PLoS One. 2015 Aug 21;10(8):e0136319. doi: 10.1371/journal.pone.0136319 (PMC4546650; doi:10.1371/journal.pone.0136319)
Supplement: S8 Table — (PDF) [file pone.0136319.s010.pdf]

S8 Table. Antagonistic associations of rs2479409 with total cholesterol (TC)

Sample	Sex	TC units	Cohort	Beta	SE	p
Nature	Men&Women	TC		1.96	0.24	3.8E-24
FHS	Men&Women	TC	C1C2C3older	1.03	0.58	7.8E-02
	Men&Women	TC	C3younger	-3.96	1.15	5.8E-04
	Men&Women	$100\times\log_{10}(TC)$	C1C2C3older	0.24	0.12	4.3E-02
	Men&Women	$100\times\log_{10}(TC)$	C3younger	-0.88	0.26	8.4E-04
	Men	$100\times\log_{10}(TC)$	C1C2C3older	0.15	0.18	4.0E-01
	Men	$100\times\log_{10}(TC)$	C3younger	-0.89	0.41	3.1E-02
	Women	$100\times\log_{10}(TC)$	C1C2C3older	0.36	0.16	2.5E-02
	Women	$100\times\log_{10}(TC)$	C3younger	-0.89	0.33	8.0E-03

"Nature" indicates the estimate from the Nature meta-analysis. "FHS" indicates the estimates from the analyses using the FHS data.
Sign of beta indicates direction of the effect in additive genetic model with minor allele considered as an effect allele, e.g., plus sign implies increasing TC values for minor allele carriers.
Demographic cohorts included men and/or women from different generations as denoted in column "Cohort", i.e., letter "C" denotes cohort with the number indicating: 1=the FHS original cohort, 2=FHSO cohort, and 3=the FHS 3rd Generation cohort. The FHS 3rd Generation cohort was stratified into younger and older sub-cohorts as defined by median cut-off for the age at biospecimens collection of 40 years. Sex is given in column "Sex".
SE denotes standard error
